# A 3D MOF based on Adamantoid Tetracopper(II) and Aminophosphine Oxide Cages: Structural Features and Magnetic and Catalytic Properties

**DOI:** 10.1021/acs.inorgchem.1c00868

**Published:** 2021-06-13

**Authors:** Ewelina I. Śliwa, Dmytro S. Nesterov, Marina V. Kirillova, Julia Kłak, Alexander M. Kirillov, Piotr Smoleński

**Affiliations:** † Faculty of Chemistry, University of Wrocław, F. Joliot-Curie 14, 50-383 Wrocław, Poland; ‡ Centro de Química Estrutural and Departamento de Engenharia Química, Instituto Superior Técnico, Universidade de Lisboa, Av. Rovisco Pais, 1049-001 Lisbon, Portugal; § Research Institute of Chemistry, Peoples’ Friendship University of Russia (RUDN University), 6 Miklukho-Maklaya st., Moscow 117198, Russian Federation

## Abstract

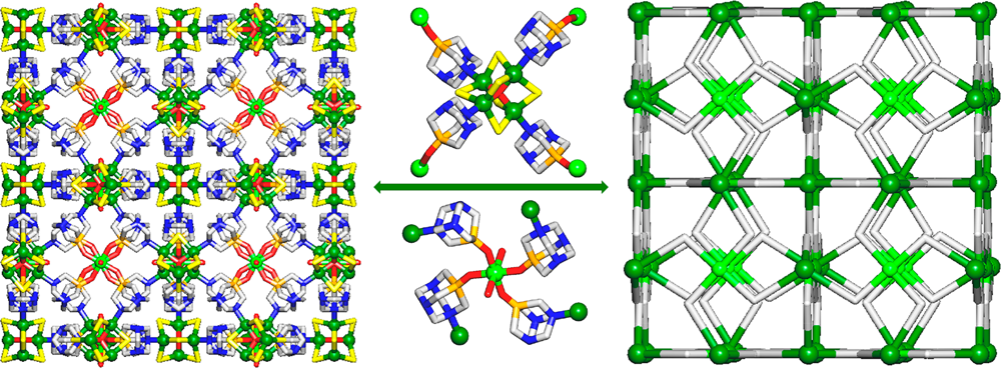

This work describes an unexpected generation of a new 3D metal–organic framework (MOF), [Cu_4_(μ-Cl)_6_(μ_4_-O)Cu(OH)_2_(μ-PTA=O)_4_]_*n*_·2*n*Cl-EtOH·2.5*n*H_2_O, from copper(II) chloride and 1,3,5-triaza-7-phosphaadamantane 7-oxide (PTA=O). The obtained product is composed of diamandoid tetracopper(II) [Cu_4_(μ-Cl)_6_(μ_4_-O)] cages and monocopper(II) [Cu(OH)_2_] units that are assembled, via the diamandoid μ-PTA=O linkers, into an intricate 3D net with an **nbo** topology. Magnetic susceptibility measurements on this MOF in the temperature range of 1.8–300 K reveal a ferromagnetic interaction (*J* = +20 cm^–1^) between the neighboring copper(II) ions. Single-point DFT calculations disclose a strong delocalization of the spin density over the tetranuclear unit. The magnitude of exchange coupling, predicted from the broken-symmetry DFT studies, is in good agreement with the experimental data. This copper(II) compound also acts as an active catalyst for the mild oxidation and carboxylation of alkanes. The present study provides a unique example of an MOF that is assembled from two different types of adamantoid Cu_4_ and PTA=O cages, thus contributing to widening a diversity of functional metal–organic frameworks.

## Abstract 2

A unique 3D MOF composed of adamantoid tetracopper(II) and aminophosphine oxide blocks was assembled and fully characterized, and its magnetic and catalytic properties were investigated.

## Introduction

Over the last decades, the synthesis of metal–organic frameworks (MOFs) has seen a tremendous development with fascinating applications in catalysis,^1,2^ magnetism,^3^ biochemistry,^4^ and materials science.^5^ Among different transition-metal compounds, copper-based MOFs are particularly attractive, given the recognized significance of copper in molecular magnetism^6^ and catalysis^7^ and its presence in the active centers of different oxidation enzymes.^8^ Hence, a good number of different bioinspired copper coordination compounds have been designed and applied in diverse catalytic transformations,^9−11^ which also include the oxidative functionalization of alkanes (very abundant but inert hydrocarbons).^7,11^ Cu-based MOFs with intriguing magnetic properties and related applications have also been reported.^6,12−14^

To build new MOFs with unique structures and functional properties, the selection of appropriate organic linkers is important. Among a variety of organic linkers applied in MOF research, the cagelike aminophosphine 1,3,5-triaza-7-phosphaadamantane (PTA) and its *P*-oxide (1,3,5-triaza-7-phosphaadamantane 7-oxide, PTA=O) are very interesting building blocks that feature a diamondoid geometry and several N,P- or N,O-sites for coordination.^15−17^ Nevertheless, despite their considerable use in aqueous organometallic chemistry, PTA and PTA=O are underexplored as building blocks for the design of MOFs.^15−17^ This might be explained by a difficulty in realizing multiple N,P- or N,O-coordination modes of PTA or PTA=O cages, respectively.^18−20^ Therefore, the use of PTA=O as a water-soluble and stable building block offers a prospective way toward the preparation of novel and structurally unique metal–organic architectures.

Following our research lines on the exploration of PTA and its derivatives in the design of new metal–organic architectures^18−22^ and investigation of their functional properties,^23−28^ we report herein the synthesis, reaction intermediate, full characterization, structural and topological features, DFT calculations, as well as the magnetic and catalytic properties of a new 3D copper(II) MOF, [Cu_4_(μ-Cl)_6_(μ_4_-O)Cu(OH)_2_(μ-PTA=O)_4_]_*n*_·2*n*Cl-EtOH·2.5*n*H_2_O (**2**).

## Results and Discussion

### Synthesis

**Scheme 1:**
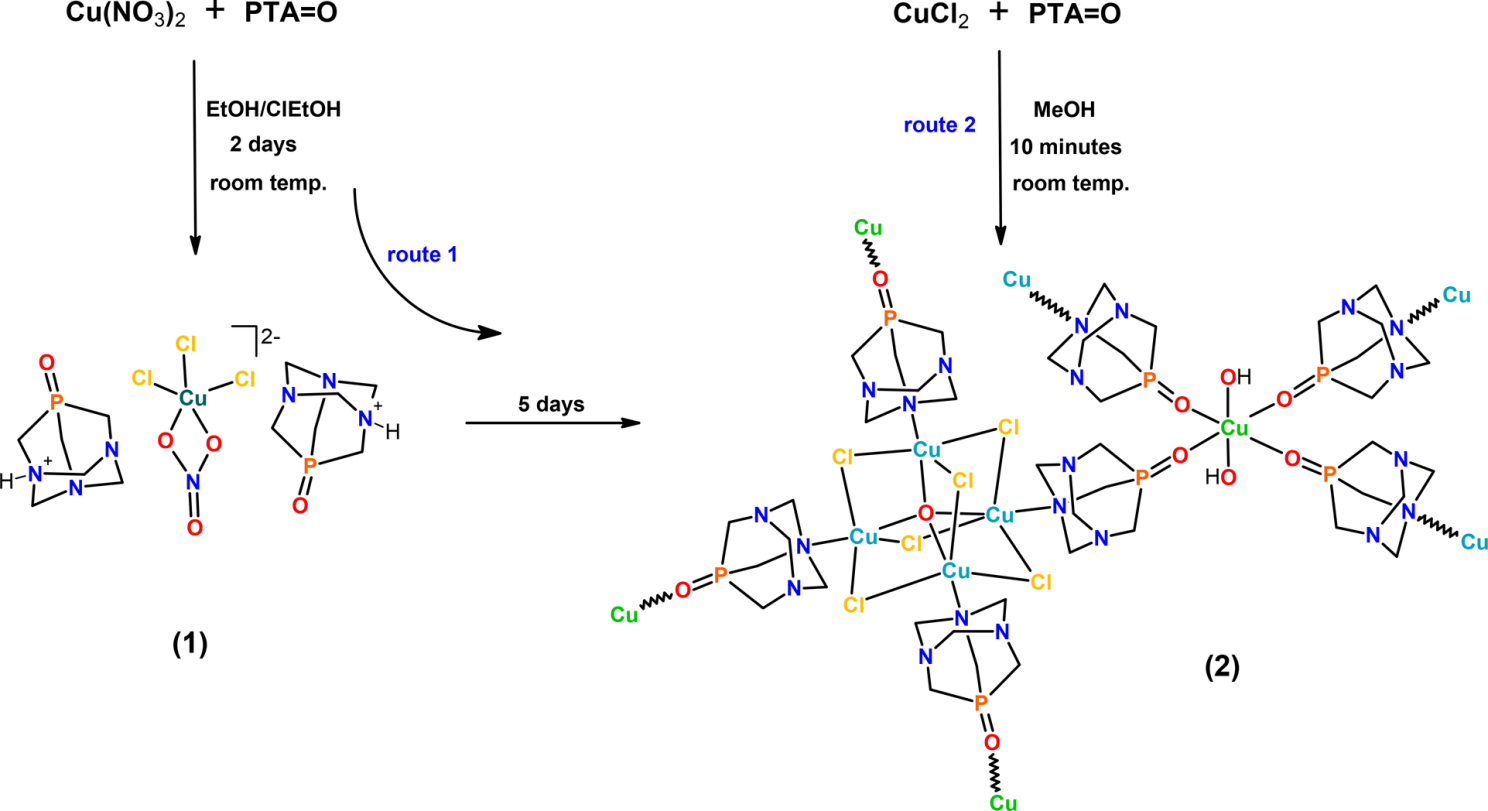
Simplified Synthetic Procedures of Compounds **1** and **2**

The compound [Cu_4_(μ-Cl)_6_(μ_4_-O)Cu(OH)_2_(μ-PTA=O)_4_]_*n*_·2*n*Cl-EtOH·2.5*n*H_2_O (**2**) was initially obtained by a facile self-assembly reaction between copper(II) nitrate and PTA=O in 2-chloroethanol/ethanol (v/v, 1/1) (route 1, Scheme 1). It was isolated as a red air-stable crystalline solid and then characterized by IR and EPR spectroscopy, and elemental, thermal, and X-ray diffraction analyses. In addition, in the course of the synthesis of **2**, the formation of the ionic monocopper(II) intermediate [H-PTA=O]_2_[CuCl_3_(NO_3_)] (**1**) was observed ([H-PTA=O]^+^ is a protonated form of PTA=O).

This hybrid inorganic–organic compound is considered as a precursor of **2**, formed via a Cu-catalyzed dechlorination of 2-chloroethanol (solvent component).^29−33^ Certainly, the source of chloride ions in compound **2** is 2-chloroethanol. The use of this chlorinated solvent is essential for the synthesis of **1** and **2**, as these products are not generated in similar reactions starting from CuCl_2_ and PTA=O in ethanol. Surprisingly, a similar reaction of CuCl_2_ and PTA=O in methanol instead of an ethanol/2-chloroethanol mixture leads to an amorphous, fine red powder of **2′** that has a different solvate system (route 2, Scheme 1). The synthetic protocols for both **2** and **2′** were optimized, maintaining the same molar copper/PTA=O ratios. Unlike the synthesis of **2′** where methanol was used as a solvent, the preparation of **2** requires a mixture of ethanol and 2-chloroethanol.

**Figure 1:**
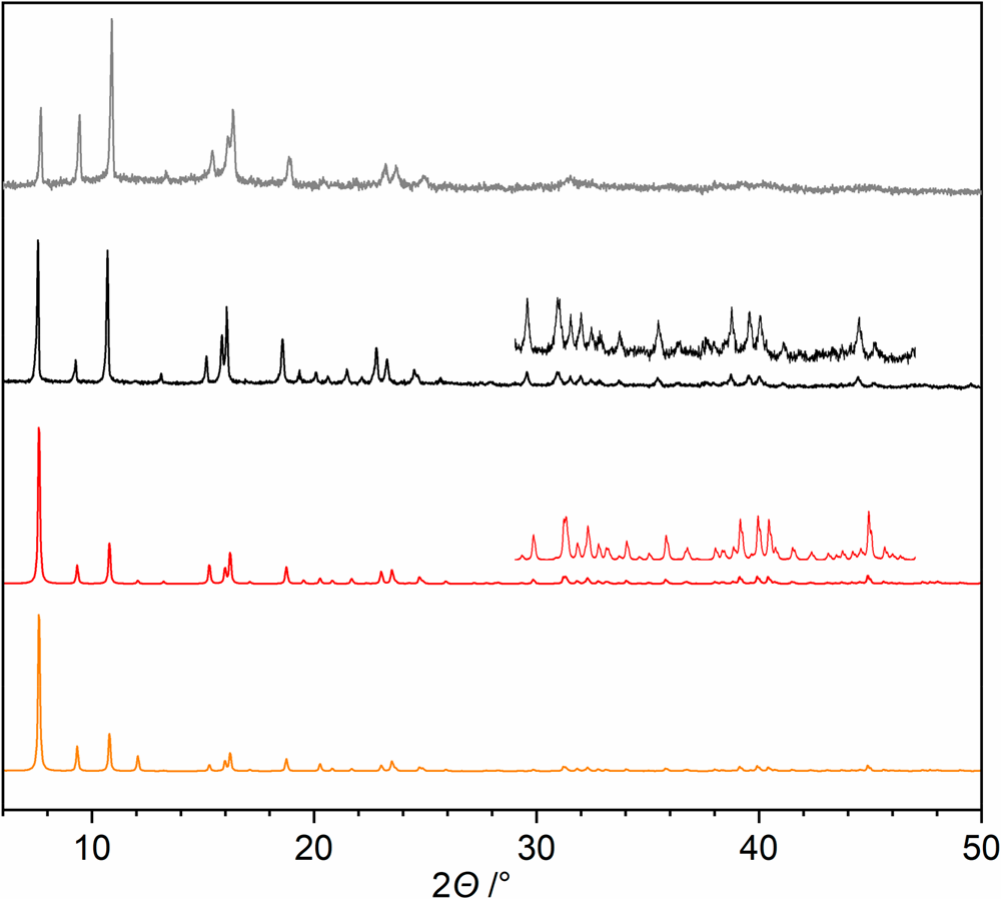
Powder X-ray patterns for **2** (route 1) and **2′** (route 2). From the top down: experimental patterns for **2′** (gray) and **2** (black) and calculated patterns for **2** (red) and **2** after removal of solvent molecules (orange). The high-angle reflections for **2** (black and red) are shown in detail.

The presence of different solvent molecules in the crystal lattice does not change the PXRD patterns of the **2** and **2′** samples obtained via routes 1 and 2. In addition, a diffractogram simulated for **2** from single-crystal X-ray data and after removal of solvent molecules shows a good match with the experimental PXRD patterns (Figure 1). For further studies, crystalline samples of **2** obtained by route 1 were used. Compound **1** was isolated as an orange crystalline solid and structurally characterized, revealing an unprecedented type of [CuCl_3_(NO_3_)]^2–^ anion, as confirmed by a search of the CSD (Cambridge Structural Database).^34^ Interestingly, in both **1** and **2**, the copper centers are five-coordinate and are simultaneously bound by three Cl^–^ ligands. The presence of hydroxo and oxo ligands in **2** is associated with hydrolysis of the aqueous Cu(II) nitrate or chloride starting materials,^35^ which might be accelerated by the presence of base (PTA=O) and alcohol solvent medium (EtOH for **2**; MeOH for **2′**). Such a hydrolysis results in the formation of Cu(OH)_2_ and Cu(μ_4_-O) fragments that are supported by the coordination of other ligands present in the reaction systems.

### Structural Description

**Figure 2:**
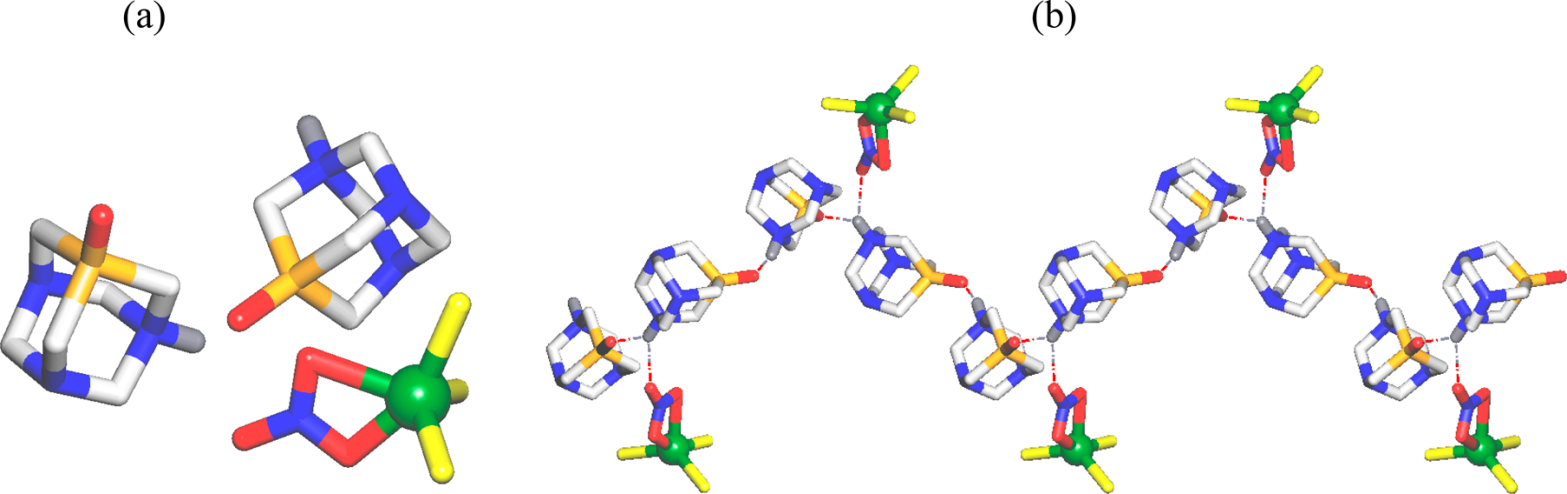
Fragments of the crystal structure of [H-PTA=O]_2_[CuCl_3_(NO_3_)] (**1**): (a) molecular unit; (b) H-bonded 1D helical chain. H atoms (except NH) are omitted for clarity. Color code: Cu (green balls), Cl (yellow), N (blue), O (red), P (orange), C (pale gray), H (gray).

The hybrid inorganic/organic structure of the intermediate [H-PTA=O]_2_[CuCl_3_(NO_3_)] (**1**) is composed of an inorganic copper(II) anion, [CuCl_3_(NO_3_)]^2–^, and two organic [H-PTA=O]^+^ cations (Figure 2a). Within the anion, the five-coordinate Cu1 atom features a significantly distorted trigonal bipyramidal {CuCl_3_O_2_} geometry that is filled by three terminal Cl^–^ ligands and a terminal bidentate NO_3_^–^ moiety (Cu1–Cl 2.227(2)–2.263(2) Å; Cu1–O 1.881(10)–2.322(12) Å). In addition, the crystal-packing pattern of **1** reveals an assembly of cations and anions via N–H···O hydrogen bonds (including a bifurcated one) into a helical 1D H-bonded chain (Figure 2b). Interestingly, despite the structural simplicity and the common combination of chloride and nitrate ligands, the present type of the anion [CuCl_3_(NO_3_)]^2–^ is unprecedented.

**Figure 3:**
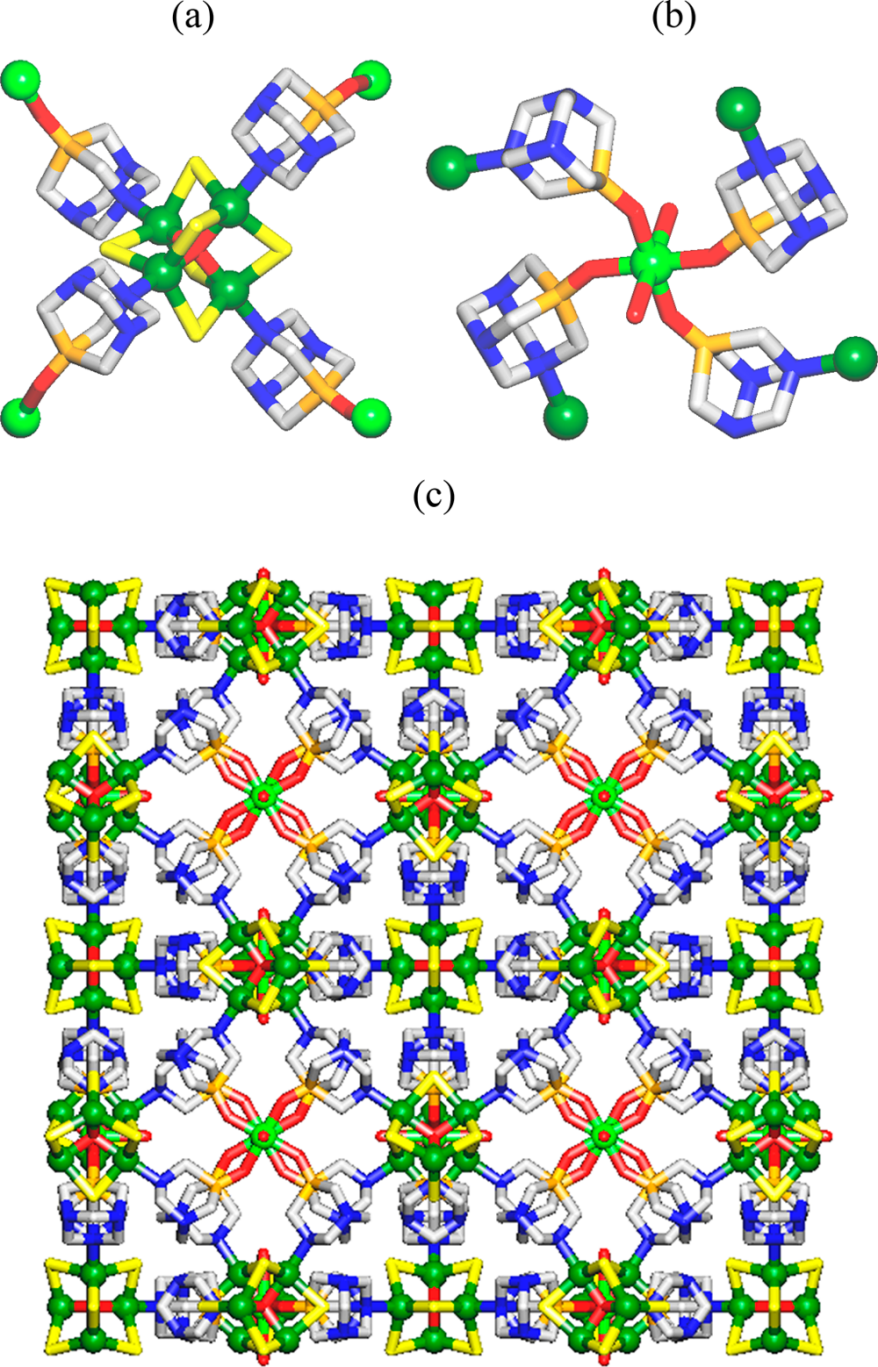
Fragments of the crystal structure of [Cu_4_(μ-Cl)_6_(μ_4_-O)Cu(OH)_2_(μ-PTA=O)_4_]_*n*_·2*n*Cl-EtOH·2.5*n*H_2_O (**2**): (a) coordination environment and connectivity of Cu1 centers (green balls) within the tetracopper(II) [Cu_4_(μ-Cl)_6_(μ_4_-O)] cage; (b) coordination environment and connectivity of Cu2 centers (light green) within the monocopper(II) [Cu(OH)_2_] moiety; (c) 3D metal–organic framework (view along the *a* axis). H atoms and solvent molecules are omitted for clarity. Color code: Cu1 (green balls), Cu2 (light green balls), Cl (yellow), N (blue), O (red), P (orange), C (pale gray).

The structure of [Cu_4_(μ-Cl)_6_(μ_4_-O)Cu(OH)_2_(μ-PTA=O)_4_]_*n*_·2*n*Cl-EtOH·2.5*n*H_2_O (**2**) discloses a very intricate 3D metal–organic framework that is driven by the tetracopper(II) adamantoid-like [Cu_4_(μ-Cl)_6_(μ_4_-O)] secondary building units (SBUs), the monocopper(II) [Cu(OH)_2_] blocks, and the μ-PTA=O linkers (Figure 3). The compound crystallizes in a cubic crystal system, and its formula unit is composed of four symmetry-quivalent Cu1 atoms, six μ-Cl^–^ ligands, one central μ_4_-O^2–^ moiety, and four equivalent μ-PTA=O linkers, in addition to a second Cu2 center with two terminal OH ligands and crystallization solvent molecules.

Within the tetracopper(II) [Cu_4_(μ-Cl)_6_(μ_4_-O)] SBU (Figure 3a), the Cu1 atoms are five-coordinate and adopt a trigonal-bipyramidal {CuCl_3_NO} geometry. It is populated by three μ-Cl^–^ ligands (Cu1–Cl 2.390(2) Å), one μ_4_-O^2–^ ligand (Cu1–O 1.9165(8) Å), and a N donor of the μ-PTA=O linker (Cu1–N 2.014(6) Å). The metal centers and ligands within the Cu_4_ SBUs are arranged into a highly symmetric adamantoid cage with Cu1···Cu1 contacts of ∼3.13 Å. Within the monocopper(II) [Cu(OH)_2_] block (Figure 3b), the Cu2 center is six-coordinate and shows an ideal octahedral {CuO_6_} geometry, which is occupied by the four O donors of the μ-PTA=O linkers (Cu2–O 1.935(7) Å) and the two terminal OH ligands (Cu2–O 2.544(7) Å). The μ-PTA=O linkers act as bidentate N,O-bridging ligands and multiply interconnect the Cu_4_ SBUs with the [Cu(OH)_2_] blocks (Cu1···Cu2 separation 6.718 Å) to generate an intricate 3D metal–organic framework (Figure 3c). Interestingly, both Cu_4_ SBUs and μ-PTA=O linkers feature an adamantoid geometry.

**Figure 4:**
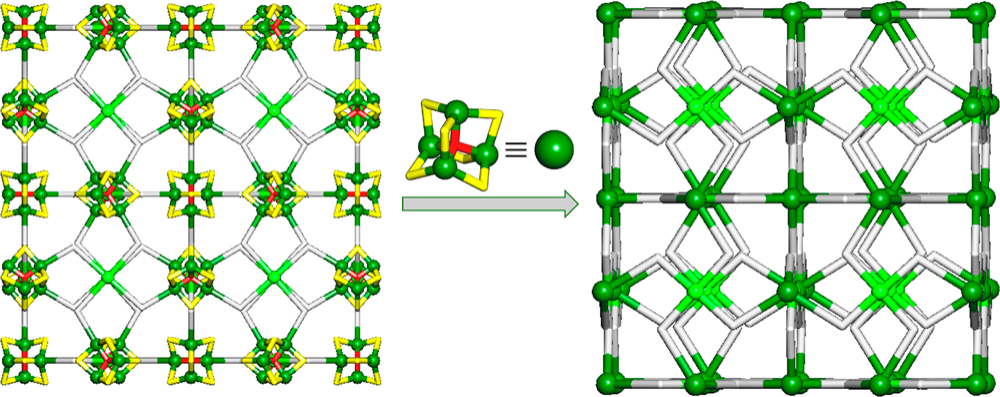
Topological simplification of the 3D MOF structure of **2**: (a) underlying net showing the connectivity of the 4-connected [Cu_4_(μ-Cl)_6_(μ_4_-O)] SBUs (Cu1, green balls; Cl, yellow; O, red) and [Cu(OH)_2_] nodes (Cu2, light green balls) through μ-PTA=O linkers (gray sticks); (b) further simplified uninodal 4-connected net with a **nbo** (NbO) topology obtained after treating the [Cu_4_(μ-Cl)_6_(μ_4_-O)] SBUs as 4-connected cage nodes (centroids of Cu_4_ SBUs, green balls). Views are along the *a* axis.

To get further insight into the structure of MOF **2**, we carried out its topological analysis by applying the concept of an underlying net.^36^ After first round of simplification (Figure 4a), all of the bridging ligands were reduced to centroids. Further simplification of this net was performed by treating the Cu_4_ SBUs as 4-connected cluster nodes (Figure 4b). These nodes, along with the 4-connected Cu2 nodes and the 2-connected μ-PTA=O linkers, form a uninodal 4-connected framework with an **nbo** (NbO) topology and point symbol of (6^4^.8^2^) (Figure 4b).

### Magnetic Studies

**Figure 5:**
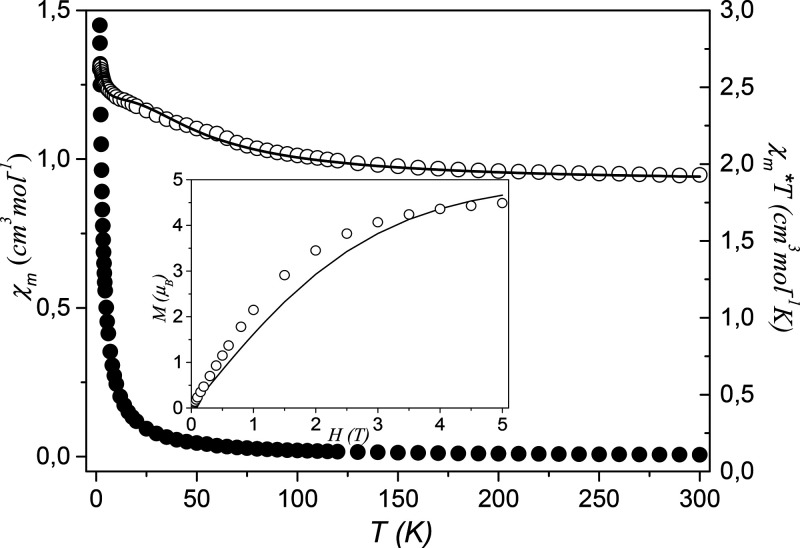
Temperature dependence of experimental χ_m_ and χ_m_*T* (χ_m_ per five Cu^II^ atoms) for **2**. The solid line is the calculated curve derived from eqs 1–5. The inset shows the field dependence of the magnetization (*M* per five Cu^II^ atoms) for **2**. The solid line is the Brillouin function curve for the system of five uncoupled spins with *S* = 1/2 and *g* = 2.0.

The magnetic properties of **2** were investigated over the 1.8–300 K temperature range. Plots of magnetic susceptibility *χ*_m_ and *χ*_m_*T* product vs *T* (*χ*_m_ is the molar magnetic susceptibility for five Cu^II^ ions) are given in Figure 5. The magnetic susceptibility of **2** increases with cooling, together with a simultaneous systematic increase in *χ*_m_*T* from 1.93 cm^3^ K mol^–1^ (3.93 μ_B_) at 300 K to 2.62 cm^3^ K mol^–1^ (4.58 μ_B_) at 1.8 K. Following prior data on Cu^II^ tetramers with the general formula Cu_4_OX_6_L_4_ (X = halogen, L = ligand),^37−45^ the *μ*_eff_ vs *T* pattern allowed the classification of such compounds into two groups: (i) those that exhibit increasing *μ*_eff_ with decreasing temperature^41−44^ and a maximum followed by a rapid decrease as the temperature is lowered further and (ii) those for which *μ*_eff_ continuously decreases on lowering the temperature. The increase in *χ*_m_*T* (*μ*_eff_) upon cooling indicates that the interactions between the copper(II) ions are ferromagnetic. The values of the Curie and Weiss constants determined from the relation *χ*_m_^*–*^^1^ = *f*(*T*) over the 1.8–300 K temperature range are equal to 1.92 cm^3^ mol^–1^ K and 3.2 K, respectively. The positive value of the Weiss constant also confirms the occurrence of ferromagnetic interactions between the copper(II) centers in **2**. To confirm the nature of the ground state of **2**, we investigated the variation of the magnetization, *M*, with respect to the field at 2 K. The results are shown in Figure 5 (see inset), where the molar magnetization *M* is expressed in μ_B_. The compound does not reach the saturation in the applied field range, and the magnetization at 5 T is equal to 4.50 μ_B_. The magnetization data were compared with the sum of the Brillouin function of five isolated copper(II) ions. The experimental values for **2** are slightly greater than that expected for five independent *S* = 1/2 systems. These results also confirm the ferromagnetic coupling between the neighboring copper(II) ions.

The structure of **2** is composed of tetracopper(II) [Cu_4_(μ-Cl)_6_(μ_4_-O)] cages and monocopper(II) [Cu(OH)_2_] units assembled into a 3D metal–organic framework. The Cu1···Cu1 separations in the {Cu_4_OCl_6_} core are approximately 3.13 Å, and the closest Cu···Cu distance between the Cu_4_ unit and Cu2 atom is 6.718 Å. A significantly weaker intermolecular interaction is expected in comparison to an intramolecular coupling within tetracopper(II) units. The magnetic susceptibility of **2** was fitted according to eq 1, as the sum of two independent contributions, namely one due to the tetracopper(II) blocks (Cu1)_4_ (*χ*_m(Cu4 unit)_) and one due to the isolated copper(II) site Cu2 (*χ*_m(Cu2 atom)_), in addition to a possible temperature-independent term (*χ*_*Nα*_), with a typical value for the copper(II) ion of 60 × 10^–6^ cm^3^ mol^–1^.
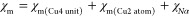
1The magnetic susceptibility per tetracopper(II) unit, *χ*_*m(Cu4 unit)*_, derived from the van Vleck formula assuming an equal *g* value for the four copper(II) ions,^46,47^ is given by eq 2
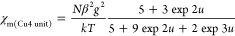
2
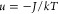
3where *J* is the intracage exchange parameter. The other symbols have their usual meaning. The contribution *χ*_m(Cu2 atom)_ is expected to follow a Curie–Weiss model for the *S* = 1/2 spins (eq 4).
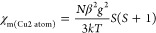
4

When the possible presence of intermolecular exchange is taken into account, eq 1 should be modified by including a molecular field correction term (*zJ’*).^48^ This yields the eq 5:
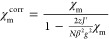
5

A least-squares fitting of the experimental data leads to the following values for **2**: *J* = +20.1(1) cm^–1^, *zJ′* = 0.04(1) cm^–1^, and *g* = 2.20(2) (*R* = 8.31 × 10^–4^). The criterion applied to determine the best fit was based on the minimization of the sum of squares of the deviation: *R* = ∑(χ_exp_*T* – χ_calc_*T*)^2^/∑(χ_exp_*T*)^2^. The calculated curve for **2** (solid line in Figure 5) matches well the experimental magnetic data in the whole temperature range. The obtained value of *J* indicates a ferromagnetic coupling within the tetracopper(II) cluster in **2**. Additionally, the value of z*J′* revealed a very weak magnetic interaction between tetracopper(II) and monocopper(II) units, as expected for quite large distances between copper(II) ions in the crystal lattice.

Previous studies of hydroxo-, alkoxo- and phenoxo-bridged copper(II) compounds^49^ indicate that the nature and the strength of the overall coupling in such systems can be influenced by the Cu···Cu distances and Cu–O–Cu angles. Generally, the longer the Cu···Cu distance, the weaker the exchange interaction. When the Cu–O–Cu angle is <97.5°, ferromagnetic interactions can be expected, whereas for angles higher than 97.5° the interaction is mostly antiferromagnetic, with an increasing magnitude as the angle increases.^49^ Therefore, the ferromagnetic coupling in **2** seems to be surprising at first glance because the Cu–O–Cu angle is higher than 109° and should cause an antiferromagnetic coupling.^49^ The structure of **2** reveals that copper(II) ions are additionally linked by chloride bridges. However, no simple magnetostructural relationship was established relating the value of the magnetic exchange constant *J* to the Cu–Cl–Cu bonding angle or Cu–Cl or Cu···Cu distances in chloro-bridged copper(II) systems.^50^ This may be due to the large variation in structural features observed, such as a number of distinct coordination geometries, which involve different orbitals in the exchange pathway. On the basis of the theory of superexchange^51^ and on the behavior of coupling parameters with bridging angles for some μ-hydroxo-copper(II) compounds,^52^ the smaller Cu–Cl–Cu angle and the shorter Cu–Cu distance may disclose a ferromagnetic interaction in **2**. Although the Cu–Cl–Cu angles and Cu···Cu distances are the most crucial geometrical parameters, the coupling constant can also be modulated by terminal ligands. The presence of N-donor apical ligands may also favor ferromagnetic coupling.^41,53^ However, slight differences in structural features and 3D structure in the case of **2** may also cause some distinction in the magnetic properties from related complexes involving the {Cu_4_OCl_6_} core. Moreover, the sign and magnitude of the *J* value obtained from the magnetic calculations for **2** match the theoretical results (DFT).

### EPR Spectra

The EPR spectra of a solid sample of **2** recorded in the X-band at room temperature and 77 K are essentially similar and additionally confirm the properties detected by the direct magnetic measurements (Figure S3). In comparison with the spectrum at 77 K, the signals at 293 K are much sharper and stronger. The observed EPR spectrum is the average pattern of four copper(II) centers in the ligand field of a trigonal-bipyramidal {CuCl_3_NO} symmetry and one in an octahedral{CuO_6_} geometry.^54−57^ Moreover, the Δ*M*_S_ = 2 transition was detected in a low-field part of the spectrum, which confirms the exchange interaction in the tetracopper(II) [Cu_4_(μ-Cl)_6_(μ_4_-O)] cage. The spectra of **2** also display a poorly resolved line at 3200 G that can be attributed to monocopper(II) [Cu(OH)_2_] units (*g* = 2.08) (Figure S3).

### DFT Calculations for Magnetic Properties of **2**

Broken-symmetry DFT calculations were performed to evaluate the exchange couplings in **2**. The calculation methodology was first tested on a series of reported binuclear copper complexes with a {Cu(μ-Cl)(μ-O)Cu} motif. These calculations showed a sufficient level of matching between experimental and calculated *J* values (Table S1) for the cases where the dimeric molecules are sterically isolated and do not contain volatile ligands (e.g., CH_3_OH or CH_3_O^–^). The difference between the experimental and calculated exchange couplings for the cases with μ-OCH_3_ could be understood if one considers alteration of the core structure during the sample preparation (e.g., grinding). A similar observation was recently made for a tetranuclear nickel complex, [Ni_4_(μ_3_-OCH_3_)_4_(Piv)_4_(CH_3_OH)_4_] (HPiv = pivalic acid), for which the magnetic properties were modeled by DFT calculations by considering the structure alteration after elimination of coordinated methanol molecules.^58^

**Figure 6:**
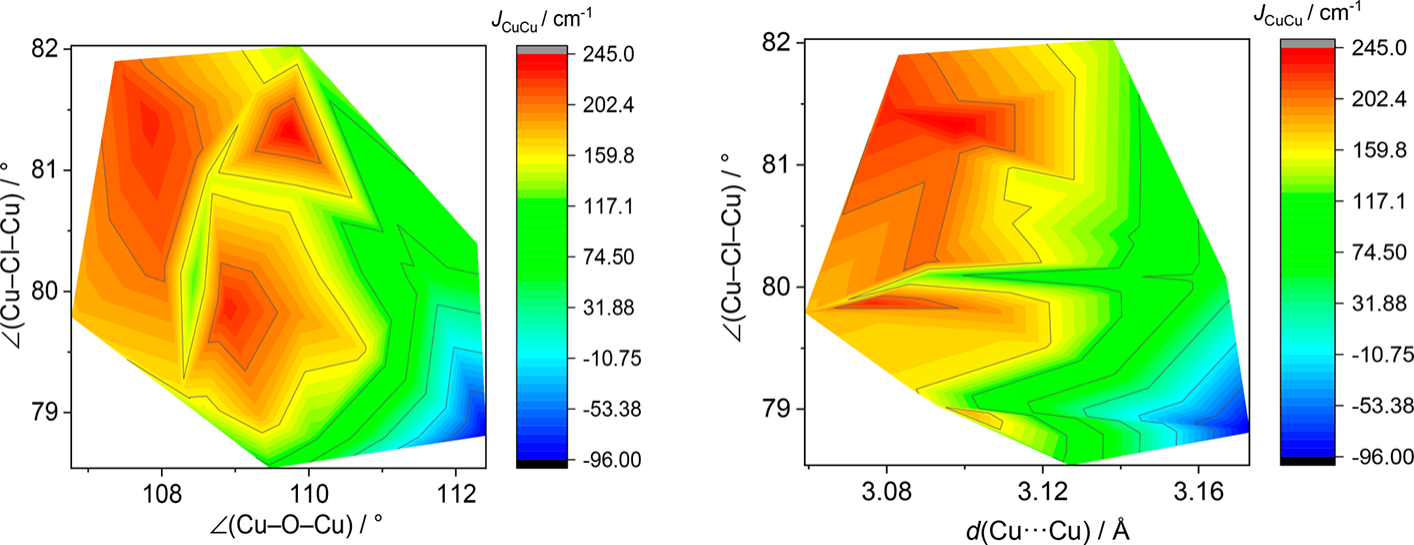
2D contour maps showing the dependences of the DFT DSM calculated exchange couplings in a {Cu^II^_4_OCl_6_X_4_} fragment (see Table S2 for details) on selected structural parameters, where two Cu^II^ atoms are replaced with Zn^II^ atoms.

The diamagnetic substitution method (DSM), where certain paramagnetic sites are replaced with diamagnetic sites (Cu^II^ → Zn^II^ in our case), was applied to evaluate the magnetic coupling between the certain Cu^II^ pairs in **2**. The DSM is known to correctly estimate the exchange couplings in polynuclear clusters,^59−61^ including very large ones, such as the Fe_34_ core.^62^ The coordination geometry of the {Cu_4_OCl_6_} core in **2** discloses two slightly different Cu···Cu separations of 3.126 and 3.138 Å in a [4 + 2] configuration. Hence, we attempted to calculate two *J* constants belonging to these separations. The {Cu_4_OCl_6_(PTAO)_4_} fragment was used, where the copper atoms as well as the atoms of the first coordination sphere were modeled at the def2-TZVPP level and all other atoms at the def2-SVP level. The results of the DSM calculations are summarized in Table S2, where three reported {Cu_4_OCl_6_} complexes (CSD refcodes CUQFID, JIWKAB, and WEXYON)^63−66^ were also used for comparative purposes. The sign and magnitude of predicted exchange couplings strongly depend on Cu–O(Cl)–Cu angles (Figure 6), as was accounted for earlier.^63,67^ The ferromagnetic coupling is favored by a smaller Cu–O–Cu angle as well as a shorter Cu···Cu separation, while a Cu–Cl–Cu angle appears to have an opposite influence on the *J* value (Figure 6). Most of the reported structures with the {Cu_4_OCl_6_} unit reveal a symmetry lower than the ideal *T*_*d*_, where the tetranuclear core shows a slight distortion. To take this fact into account, we attempted to calculate six independent *J*_CuCu_ exchange integrals for a series of literature complexes bearing a {Cu_4_OCl_6_} core (Table S2).

**Figure 7:**
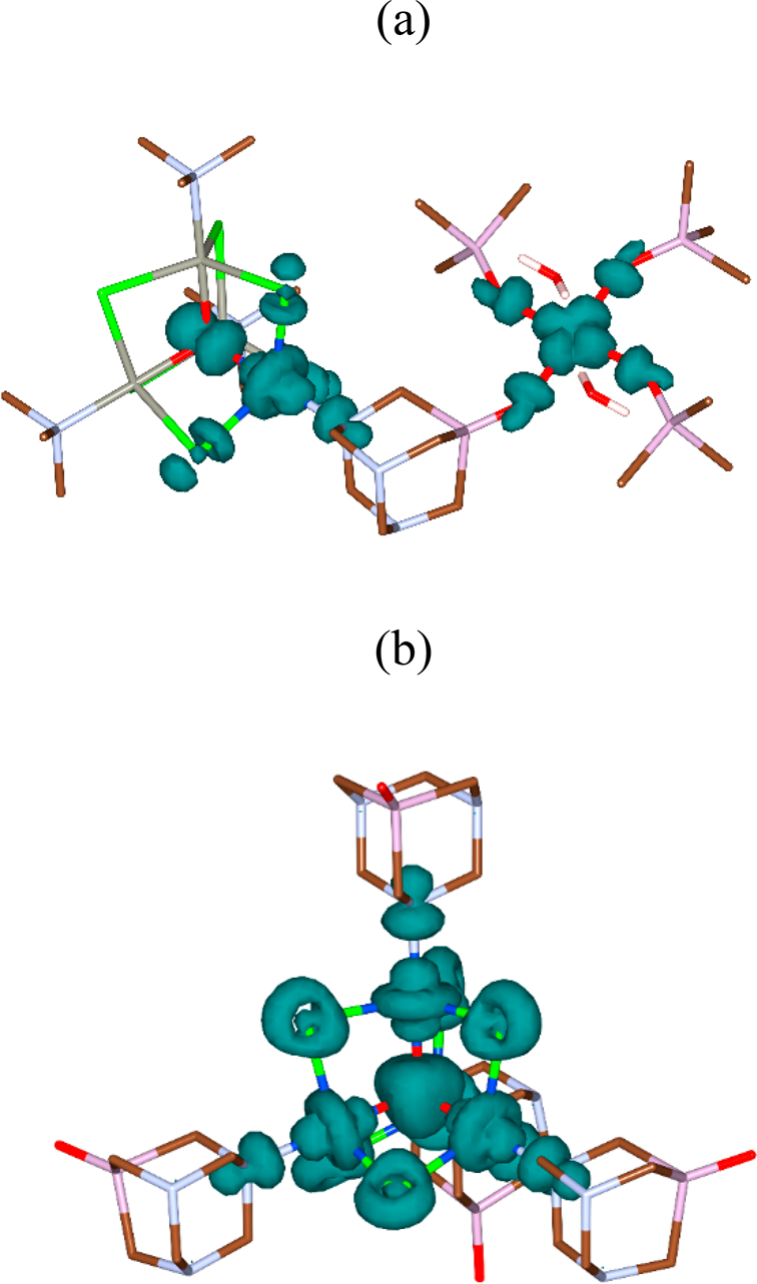
(a) Isosurface of the DFT-calculated spin density for the triplet state of {Zn_3_CuOCl_6_(NMe_3_)_3_(PTAO)Cu(Me_3_PO)_3_(H_2_O)_2_} with a cutoff value of 0.003 e *a*_0_^3^. The respective fragment was obtained by truncating the PTAO ligands in the structure of **2** and replacing three copper sites with zinc sites. (b) Isosurface of the DFT-calculated spin density for the quintet state of {Cu_4_OCl_6_(PTAO)_4_} (a fragment of the structure of **2**) with the same cutoff value. The hydrogen atoms (except those of water ligands) are eliminated for clarity. Color code: Cu, blue; Zn, gray; Cl, green; O, red; N, light blue; P, purple; C, brown; H, white.

The interaction between the “isolated” copper site Cu2 and the closest copper site from the Cu_4_ unit was predicted to be weakly antiferromagnetic (Table S2 and Figure 7). Although the closest Cu···Cu distance between the Cu_4_ unit and Cu2 atom is 6.718 Å, it is known that the couplings at a comparable distance could be quite strong (up to 35 cm^–1^).^68^ In the case of **2**, the calculations evidence that the superexchange interaction mediated by the PTA=O ligand is weak but is not zero (Table S2).

The predicted magnitudes of exchange couplings using the DSM model are considerably higher than the experimental values (Table S2). The explanation of this inconsistency lies in strong spin delocalization within the {Cu_4_OCl_6_} cluster, which disables the possibility of a correct determination of the separate *J* integrals by DSM. With two copper atoms replaced with zinc atoms, the main spin density lies on the remaining copper atoms (Table 1). The spin population on bridging Cl and O atoms is considerably smaller, as expected for a typical Cu–X–Cu superexchange. However, the single-point calculations for a quintet state of {Cu_4_OCl_6_} fragments in various complexes reveal the spin density on a central μ_4_-O atom to be comparable to that for copper atoms (Table 1 and Figure 7). The effect of pronounced spin delocalization in {Cu_4_OCl_6_X_4_} complexes^69^ was experimentally confirmed recently by polarized neutron diffraction.^70^ Thus, the mutual influence of the copper shoulders is too high and the DSM model is not applicable in this case.

**Table 1:** Selected Mulliken Spin Populations for the High-Spin State of {Cu_4_OCl_6_(PTAO)_4_} and Its Derivatives with Two Copper Atoms Replaced with Zinc Atoms^a^

site	*J*_1_ (Cu3 = Zn, Cu4 = Zn)^b^	*J*_2_ (Cu2 = Zn, Cu4 = Zn)^c^	full Cu_4_
Cu1	0.572068	0.571586	0.576406
Cu2	0.572068	–0.003306	0.576405
Cu3	–0.003191	0.571586	0.576406
Cu4	–0.003191	–0.003306	0.576405
O	0.292723	0.292543	0.534758
Cl12^d^	0.116693	0.054986	0.113350
Cl23	0.055162	0.055168	0.113908
Cl34	0.009226	0.054986	0.113350
Cl14	0.055162	0.055168	0.113908
Cl13	0.055162	0.117274	0.113908
Cl24	0.055163	0.009254	0.113908
N1^e^	0.092239	0.092251	0.098293
N2	0.092240	0.000552	0.098293
N3	0.000563	0.092251	0.098292
N4	0.000563	0.000552	0.098293

a The atom coordinates are obtained from an X-ray analysis: B3LYP/G functional, def2-TZVPP basis set for metal atoms and first coordination environment, def2-SVP set for all other atoms.

b *d*(Cu1···Cu2) = 3.138 Å.

c *d*(Cu1···Cu3) = 3.126 Å.

d The numbers mean the copper atoms bridged by the respective chlorine atom.

e The numbers indicate the copper atom coordinated by the respective nitrogen atom.

**Figure 8:**
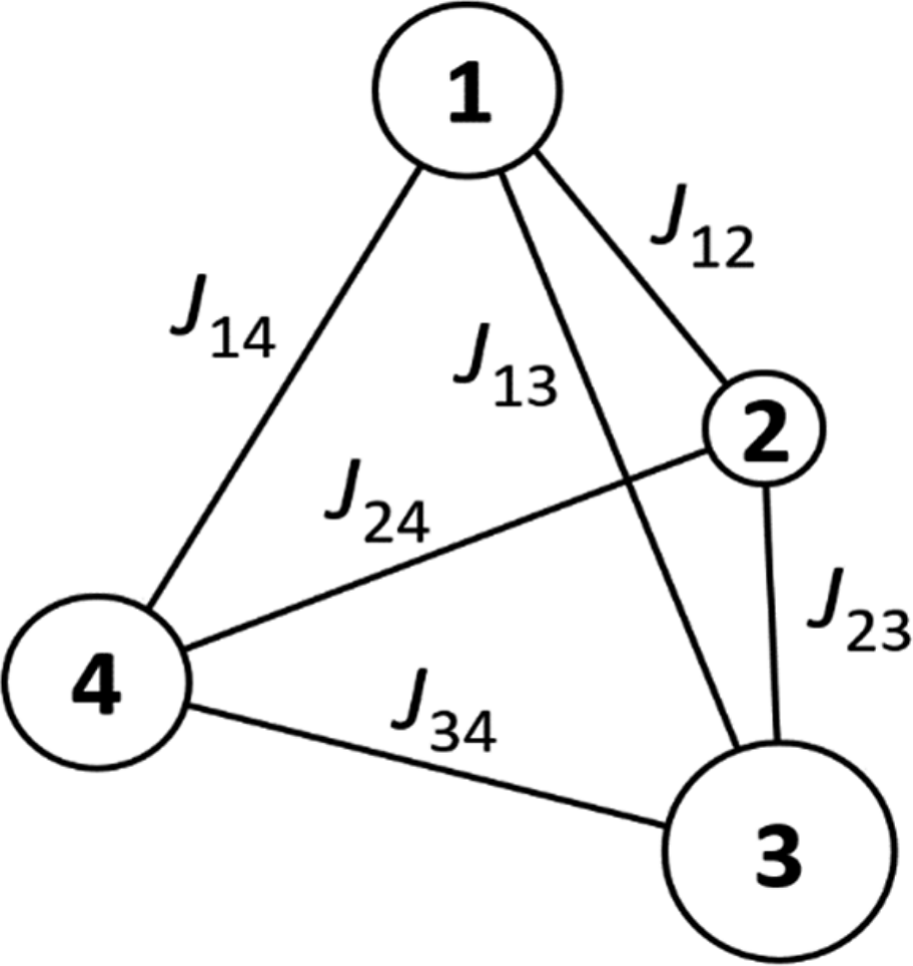
Schematic representation of the tetranuclear core in **2**, showing the numbering of the *J* constants.

The *J* integrals in a polynuclear complex could be estimated by knowing the energies of the high-spin (*E*_HS_) and broken-symmetry (*E*_BS_) states, using the approach neglecting the correction for spin projection, developed by Ruiz.^71^ In this model, *E*_BS_ – *E*_HS_ = (2*S*_1_*S*_2_ + *S*_2_)*J*_12_.^72^ For Cu^II^ ions with *S*_1_ = *S*_2_ = 1/2 this formula simplifies to *E*_BS_ – *E*_HS_ = *J*_12_. The accuracy of this approach has been confirmed by its successful use toward modeling of the exchange couplings in polynuclear complexes, including large-nuclearity cages.^73−75^ The exchange couplings in the system of four interacting spins {1,2,3,4} can be described by six exchange integrals: *J*_12_, *J*_13_, *J*_14_, *J*_23_, *J*_24_, and *J*_34_ (considering the symmetric exchange only). The reliable determination of *n* variables requires *n* + 1 equations. Therefore, the system depicted in Figure 8 requires the calculation of Δ_*ij*_ = *E*_HS_ – *E*_BS*ij*_ energy differences for at least seven broken-symmetry *ij* configurations (where the numbers *i* and *j* indicate the site(s) with flipped α → β spins).

According to the Ruiz approach, the differences Δ_*ij*_ for the model depicted in Figure 8 are expressed as
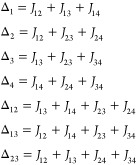
6from which the exchange coupling constants can be derived
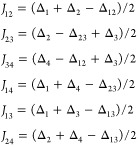
7

As the calculation of the complete {Cu_4_OCl_6_(PTAO)_4_} fragment at the def2-TZVPP level is time-consuming, we investigated the possibility of using basis sets of reduced precision, as well as the use of truncated PTA=O ligands. The respective results are summarized in Table S3. As can be seen, the nature of the substituent has a strong influence on the electron configuration of the whole molecule. The choice of a basis set was also crucial. We finally found that the {Cu_4_OCl_6_(NMe_3_)_4_} fragment (where the coordinates of N and C atoms for NMe_3_ groups are from the truncated PTA=O ligands, with generated hydrogen atoms) and def2-TZVP basis set for all atoms give a Δ_12_ energy gap similar to that for the {Cu_4_OCl_6_(PTA=O)_4_}/def2-SVP/def2-TZVPP combination in a reasonable computational time. Thus, we used the above settings for further studies.

**Figure 9:**
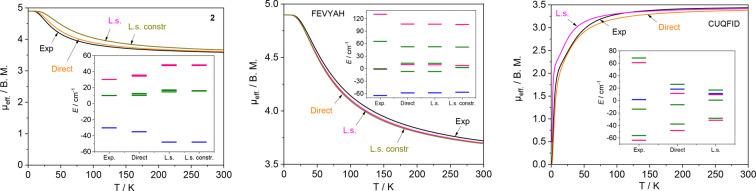
Reconstructed μ_eff_ vs *T* dependences and energy levels (color scheme for insets: singlet, red; triplet, green; quintet, blue) for the {Cu_4_OCl_6_(NMe_3_)_4_} fragment of the structure **2** (left), the [Cu_4_(NH_3_)_4_(HL^8^)_4_]^4+^ cation^76^ in the structure FEVYAH (middle), and the neutral [Cu_4_OCl_6_(L^2^)_4_] complex^63^ (CUQFID; right); see Table 4 for details. The magnetically isolated paramagnetic copper site Cu2 in **2** was not included in the simulation. All of the curves were adjusted by applying *g* = 2.0. The reported magnetic curve for FEVYAH shows the distinct decay of the magnetic moment at low temperature due to zero-field splitting,^76^ not accounted for in the present study.

The results of the respective calculations for **2** as well as two tetranuclear complexes in the literature are summarized in Table S4. We first applied this approach to the tetranuclear cubanelike cation in [Cu_4_(NH_3_)_4_(HL^8^)_4_][CdBr_4_]Br_2_·3DMF·H_2_O (CSD refcode FEVYAH; H_2_L^8^ = diethanolamine) with the *S* = 2 ground spin state.^76^ This compound does not exhibit spin delocalization, and its magnetic properties were precisely fitted with the support of a multifrequency, high-field EPR technique. By using the Ruiz approach, an overall behavior and exchange model symmetry can be predicted with sufficient precision (Table S4 and Figure 9). Next, we tested the same strategy on the fully asymmetric complex [Cu_4_OCl_6_(L^2^)_4_] (CSD refcode CUQFID) bearing a tetranuclear core resembling that in **2**.^63^ Although the predicted symmetry and distribution of the energy levels differ from those determined experimentally, the bulk magnetic behavior is correctly reproduced (Figure 9). For **2**, all of the exchange interactions were predicted to be ferromagnetic, with the symmetry very close to *T*_*d*_ assumed by the experimental model (Table S4). The difference in magnitudes between calculated and experimental couplings could be due to the simplified structural fragment used for the calculations, while the magnetic exchange it strongly dependent on the nature of the ligands in apical positions at the copper centers within a Cu_4_ unit (Table S3). Also, the featureless magnetic curve of **2** (Figure 5) prevents fitting with more than one exchange coupling constant due to the risk of overparameterization, neglecting in this way some possible slight differences between the individual coupling constants.

### Catalytic Studies

Following our interest in the metal-catalyzed oxidative functionalization of alkanes under mild conditions,^77−79^ we applied compound **2** as a catalyst for the oxidation and carboxylation of gaseous and liquid alkanes (i.e., propane and cyclohexane) to alcohols and ketones or carboxylic acids.

In contrast to numerous Cu-catalyzed systems for alkane oxidation, the reactions catalyzed by **2** do not require the presence of an acid promoter.^77−81^ The oxidation of an inert light alkane such as propane in the presence of **2** leads to a mixture of isopropyl alcohol, acetone, *n*-propanol, and propanal with a total yield of 14% based on C_3_H_8_ and a TON (turnover number; e.g. moles of product per mole of catalyst) value of up to 280 (Table 2). Isopropyl alcohol and acetone are the main products due to the more reactive nature of the secondary C atom of the propane molecule. The best TONs are observed when higher substrate pressures (loadings) are used. At a lower propane pressure, there is less substrate present in the system and thus the overall product yield is higher, although the TON values are lower (Table 2). The propane oxidation is rather quick, resulting in the maximum product yields within a few hours. The product yields observed after the 24 h reaction time slightly decrease due to partial overoxidation. The effect of the acid promoter is not significant in the oxidation of propane, leading to comparable yields (Table 2, entries 4 and 5); only a minor improvement was observed in the presence of a TFA promoter. The fact that the catalyst is active without an acid promoter represents a feature of this system. The activity achieved herein is considerable when the high inertness of gaseous propane and mild reaction conditions applied are taken into account.

**Table 2:** Mild Oxidation of Propane Catalyzed by **2**^a^

					product yield, %^b^					
entry	catalyst amount, μmol	C_3_H_8_, atm	*T*, °C	time, h	isopropyl alcohol	acetone	*n*-propanol	propanal	total	TON^c^
1	2.5	3	25	24	2.0	2.2	1.0	0.7	5.9	50
2	2.5	3	50	3	3.0	2.6	1.2	0.5	7.3	62
3	2.5	3	50	24	1.9	3.9	0.9	0.3	7.0	59
4	1.25	3	50	5	4.6	2.4	1.9	0.7	9.6	161
5	1.25^d^	3	50	5	5.1	3.1	2.1	0.6	10.9	182
6	1.25	3	50	24	2.9	5.7	1.1	0.5	10.2	170
7	1.25	1	50	5	3.9	7.0	2.1	0.8	13.8	78
8	1.25	1	50	24	2.9	8.5	1.5	0.6	13.5	76
9	1.25	8	50	5	3.0	1.4	1.4	0.4	6.2	280
10	1.25	8	50	24	2.9	1.6	1.2	0.4	6.1	274

a Reaction conditions: C_3_H_8_ (1–8 atm, 0.7–5.6 mmol), H_2_O_2_ (50% aqueous, 5.0 mmol), **2** (1.25–2.5 μmol), CH_3_CN (up to 2.5 mL total reaction volume), 50 °C in a stainless-steel autoclave (20 mL capacity).

b Yields are based on propane: (moles of product)/(mole of propane) × 100%.

c TON, turnover number: (moles of product)/(mole of catalyst).

d In the presence of TFA promoter (0.025 mmol).

The oxidation of cyclohexane catalyzed by **2** results in ∼15% yields (based on C_6_H_12_) of the products, cyclohexanol and cyclohexanone, already after 10 min of the reaction (Figures S10 and S11). The presence of 10 equiv (relative to the catalyst amount) of TFA (trifluoroacetic acid, known as an efficient promoter for Cu-containing catalytic systems) does not affect either the reaction rate or the total product yield (Figure S10). Cyclohexane is transformed to a mixture of cyclohexanol (main product) and cyclohexanone with a total yield of 17%. The reaction rate is very high, resulting in a TOF (turnover frequency, i.e. moles of products per mole of catalyst per hour) value of ∼500 h^–1^ and a total yield of 14.4% after 7 min.

We also studied the effect of the catalyst amount on the maximum initial reaction rate (*W*_0_) and the total product yield in the cyclohexane oxidation catalyzed by **2** (Figure S11). When the catalyst amount is augmented from 1.25 to 10 μmol, the *W*_0_ value increases with a nonlinear dependence. This indicates a reaction order above 1, suggesting that more than one Cu-containing catalytically active species participates in the rate-limiting reaction step. The same effect was also observed when multinuclear Fe-based catalytic systems were used for alkane oxidation.^82−84^ When the catalyst amount in the reaction solution was lowered from 10 to 1.25 μmol, the maximum TON grew from 68 to 298.

Compound **2** was also screened as a catalyst for the mild single-pot carboxylation of cyclohexane or propane to form cyclohexanecarboxylic acid or a mixture of butyric acids, respectively (Table 3). The reaction protocols involve the treatment of the alkane with CO (carbonyl source), H_2_O (hydroxyl source), and K_2_S_2_O_8_ (oxidant and radical initiator) in an aqueous acetonitrile solution at 60 °C. Thus, the carboxylation of C_6_H_12_ yields 30% of C_6_H_11_COOH, along with minor amounts of cyclohexanone (1.2%) and cyclohexanol (0.3%) as a result of partial alkane oxidation. Propane is transformed to a mixture of isobutyric (main product) and *n-*butyric acids with a total yield of 28% (Table 3). In the present work, the activity of **2** in the carboxylation of hydrocarbons^78,85^ is comparable to those of some other systems reported in our earlier studies (Table S5 in the Supporting Information).

**Table 3:** Cu-Catalyzed Hydrocarboxylation of Alkanes^a^

	yield, %^b^	
alkane	products	total^c^
cyclohexane	30.0 (cyclohexanecarboxylic acid, C_6_H_11_COOH)	31.5
	1.2 (cyclohexanone, C_6_H_10_O)	
	0.3 (cyclohexanol, C_6_H_11_OH)	
propane	22.0 (isobutyric acid, (CH_3_)_2_CHCOOH)	28.2
	6.2 (*n*-butyric acid, C_3_H_8_COOH)	

a Reaction conditions: cyclohexane (1 mmol) or propane (1 atm), CO (20 atm), **2** (2.5 μmol), CH_3_CN (4 mL), H_2_O (2 mL), K_2_S_2_O_8_ (1.5 mmol), 3 h, 60 °C, stainless-steel autoclave (20 mL capacity).

b Yields are based on alkane: (moles of product)/(mole of cycloalkane) × 100%.

c Sum of the yields of all products.

## Conclusions

In summary, we synthesized a unique 3D metal–organic framework which is driven by two different types of Cu^II^_4_ and Cu^II^ blocks and μ-PTA=O linkers. The present compound extends a very limited family of MOFs assembled from cagelike aminophosphine oxide linkers, which are still poorly used for the design of metal–organic architectures. In addition, an unusual Cu-catalyzed dechlorination of 2-chloroethanol was observed upon treatment of copper(II) nitrate and PTA=O in a mixture of 2-chloroethanol/ethanol, allowing the isolation and full characterization of a new hybrid inorganic/organic material, [H-PTA=O]_2_[CuCl_3_(NO_3_)] (**1**), which is an intermediate in the synthesis of **2**. The structural and topological features of the obtained compounds and their H-bonding or metal–organic frameworks were discussed in detail. Furthermore, the magnetic behavior of MOF **2** was investigated by different methods, including DFT calculations. Variable-temperature magnetic susceptibility and EPR studies indicate a ferromagnetic interaction between the neighboring copper(II) ions in the adamantoid Cu_4_ unit. Moreover, compound **2** acts as an efficient catalyst for the oxidation and hydrocarboxylation of alkanes under mild conditions. The present work also provides a unique example of a functional MOF that is assembled from two different types of adamantoid Cu_4_ and PTA=O cages.

## Experimental Section

### Materials and Methods

All synthetic work was performed in air. Copper(II) nitrate trihydrate (POCh) and solvents (anhydrous ethanol, 2-chloroethanol; Sigma-Aldrich) were obtained from commercial sources. 1,3,5-Triaza-7-phosphaadamantane 7-oxide (PTA=O) was synthesized in accord with literature methods.^86,87^ The infrared spectra (4000–400 cm^–1^) were recorded on a Bruker Vertex 70 FT–IR instrument using KBr pellets. Elemental analyses and thermal analyses were performed using a CHNS Vario EL CUBE apparatus and a TG-DTA Setaram SETSYS 16/18 instrument (corundum Al_2_O_3_, 100 μL crucible, N_2_ atmosphere, heating rate of 10 °C/min), respectively. Powder X-ray diffraction (PXRD) analyses were performed on a Bruker D8 ADVANCE diffractometer using Cu Kα radiation (λ = 1.5418 Å) filtered with Ni. The diffractograms were recorded with a step size of 2θ = 0.016° over the range 2θ = 5–60° and ratio 0.5. The calculated pattern was obtained from the single-crystal XRD data using the MERCURY CSD 3.9 package. In catalytic studies, gas chromatography (GC) analyses were carried out on an Agilent Technologies 7820A series gas chromatograph (detector, flame ionization; carrier gas, He; capillary column, BP20/SGE, 30 m × 0.22 mm × 0.25 μm).

### Synthesis and Analytical Data

The compounds [H-PTA=O]_2_[CuCl_3_(NO_3_)] (**1**) and [Cu_4_(μ-Cl)_6_(μ_4_-O)Cu(OH)_2_(μ-PTA=O)_4_]_*n*_·2*n*Cl-EtOH·2.5*n*H_2_O (**2**) and their single crystals were obtained by diffusing an ethanol solution (40 mL) of copper(II) nitrate trihydrate (492.6 mg, 2.0 mmol) on a 2-chloroethanol solution layer of 1,3,5-triaza-7-phosphaadamantane 7-oxide (710.5 mg, 4.0 mmol) (40 mL) in a tube (route 1). Orange crystals of **1** were found at the junction of the two solutions after 1 day and could be isolated and analyzed by X-ray diffraction (20% yield, based on copper(II) nitrate). However, if **1** is not isolated and the tube is kept for 1 week, there is a transformation of **1** into red crystals of compound **2** (10% yield, based on the copper(II) nitrate). Data for compound **1** are as follows. FT-IR (KBr, cm^–1^): 3435 (m), 3007 (m), 2962 (m), 2926 (m), 2852 (w), 2626 (w), 2345 (vw), 1734 (vw), 1635 (vw), 1484 (s), 1463 (s), 1384 (s), 1351 (m), 1293 (vs), 1275 (s), 1259 (m), 1246 (m), 1212 (s), 1164 (vs), 1108 (s), 1089 (w), 1045 (w), 1028 (s), 1016 (s), 982 (s), 955 (vs), 938 (m), 916 (w), 900 (w), 822 (s), 787 (m), 763 (m), 737 (w), 660 (w), 606 (m), 552 (m), 452 (vw), 441 (w), 417 (m), 386 (w). Compound **2** is soluble in water, DMSO, DMF, and 2-chloroethanol, slightly soluble in acetone, 1,4-dioxane, THF, and MeCN, and insoluble in MeOH, EtOH, dichloromethane, toluene, and octanol. FT-IR (KBr, cm^–1^): 3413 (m), 2960 (w), 2925 (w), 1630 (vw), 1447 (s), 1413 (m), 1384 (s), 1291 (vs), 1250 (m), 1227 (m), 1158 (vs), 1104 (m), 1085 (m), 1022 (s), 987 (s), 957 (vs), 889 (m), 848 (w), 829 (m), 799 (w), 772 (w), 742 (s), 676 (w), 655 (w), 623(s), 556 (s), 457 (w), 440 (w). Anal. Calcd for C_24_H_50_Cu_5_N_12_O_7_P_4_Cl_6_·2ClEtOH·3H_2_O (MW 1488.14): C, 22.60; H, 4.47; N, 11.29. Found: C, 22.61; H, 4.33; N, 11.30. TG-DTA for **2** (N_2_, 5 °C/min): 50–150 °C {−2ClEtOH – 3H_2_O}, Δ*m* (%; 14.49 exptl, 14.45 calcd): >150 °C (dec).

The synthesis of [Cu_4_(μ-Cl)_6_(μ_4_-O)Cu(OH)_2_(μ-PTA=O)_4_]_*n*_·6*n*H_2_O (**2′**) was as follows (route 2). To a methanol solution (2 mL) of CuCl_2_·2H_2_O (85.4 mg, 0.5 mmol) was added a methanol solution (45 mL) of PTA=O (182.9 mg, 1 mmol) dropwise. The reaction mixture was stirred at room temperature for 10 min and then filtered off, producing a fine dark red solid of **2′** (33% yield, based onCuCl_2_). FT-IR (KBr, cm^–1^): 3429 (s), 2960 (m), 2924 (m), 1643 (m), 1470 (w), 1447 (m), 1426 (w), 1413 (w), 1293 (vs), 1267 (w), 1253 (w), 1228 (m), 1156 (vs), 1106 (m), 1088 (vw), 1023 (s), 988 (m), 959 (vs), 899 (m), 830 (w), 800 (vw), 773 (w), 744 (m), 678 (w), 624 (m), 558 (m), 458 (vw), 440 (vw). Anal. Calcd for C_24_H_50_Cu_5_N_12_O_7_P_4_Cl_6_·6H_2_O (MW 1381.16): C, 20.87; H, 4.52. Found: C, 20.16; H, 4.31. TG-DTA for **2′** (N_2_, 5 °C/min): 50–100 °C {−6H_2_O} Δ*m* (%; 7.06 exptl, 7.81 calcd): >150 °C (dec).

### Refinement Details for X-ray Analysis and Crystal Data

Single-crystal data collection was performed on a KUMA Xcalibur diffractometer with a Sapphire CCD detector, equipped with an Oxford Cryosystems open-flow nitrogen cryostat, using ω scans and graphite-monochromated Mo Kα (λ = 0.71073 Å) radiation. Cell refinement, data reduction, analysis, and absorption correction were carried out with CrysAlis PRO (Rigaku Oxford Diffraction, Wrocław, Poland) software.^88^ The structures were solved by direct methods with SHELXT-2014/5 and refined with full-matrix least-squares techniques on *F*^2^ with SHELXL-2018/3.^89,90^ The void and difference electron density maps were generated with Olex2-1.3.0.^91^ The nitrate (N7, O3–O5) and chloride (Cl4) anions in **1** were modeled as being substitutionally disordered with site occupations of 0.559(11) and 0.441(11), respectively. The structure of **2** contains large voids of 1412 Å^3^ occupied by solvent molecules (Figure S1). The peaks observed in the difference Fourier map were modeled as chloroethanol and water molecules. The bond distances and angles in the chloroethanol model were restrained to ideal values. The H atoms of the water molecules were not localized. All other hydrogen atoms in **1** and **2** were placed at calculated positions and refined using the riding model with *U*_iso_ = 1.2*U*_eq_.

#### Crystal data for **1**:

C_12_H_26_Cl_3.44_CuN_6.56_O_3.67_P_2_, *M* = 568.41, *a* = 8.6028(6) Å, *b* = 15.9849(9) Å, *c* = 15.6635(15) Å, β = 90.774(9)°, *V* = 2153.8(3) Å^3^, *T* = 79.8(6) K, space group *P*2/*c*, *Z* = 4, Mo Kα, 10479 reflections measured, 4854 independent reflections (*R*_int_ = 0.0746), R1 = 0.0918 (*I* > 2σ(*I*) for 2823 reflections), wR2 = 0.1876, GOF = 1.088.

#### Crystal data for **2**:

C_28_H_65_Cl_8_Cu_5_N_12_O_11.50_P_4_, *M* = 1479.10, *a* = *b* = *c* = 32.7826(3) Å, *V* = 35231.4(10) Å^3^, *T* = 79.8(6) K, space group *Fm*3̅*c*, *Z* = 24, Mo Kα, 16174 reflections measured, 1381 independent reflections (*R*_int_ = 0.1089), R1 = 0.0581 (*I* > 2σ(*I*) for 946 reflections), wR2 = 0.1714, GOF = 1.024.

Crystallographic data for the structures reported in this paper have been deposited with the Cambridge Crystallographic Data Centre as CCDC-2064900 (**1**) and CCDC-2064901 (**2**).

### Magnetic and Electron Paramagnetic Resonance (EPR) Studies

The magnetic properties were investigated over the temperature range of 1.8–300 K on a Quantum Design MPMS3 SQUID magnetometer. Solid-state EPR spectra of **2** were recorded on a Bruker ELEXSYS E500 CW-EPR spectrometer in X-band at 298 and 77 K. The magnetization of powdered sample **2** was measured over the 1.8–300 K temperature range using a Quantum Design SQUID-based MPMSXL-5-type magnetometer. The superconducting magnet was generally operated at a field strength ranging from 0 to 5 T. Sample measurements were made at a magnetic field of 0.5 T. The SQUID magnetometer was calibrated with a palladium rod sample. Corrections are based on subtracting the sample-holder signal, and the *χ*_D_ contribution was estimated from Pascal’s constants.^92^

### DFT Calculations

“Broken-symmetry”^72,93,94^ calculations were carried out by using the B3LYP/G functional^94−98^ with the TZVPP basis set for the copper atoms and coordination sphere and SVP for all other atoms (unless stated otherwise), using the ORCA 4.2.1 package^99^ with integration grids Grid4. For some cases the chain-of-spheres RIJCOSX approximation was applied, with the support of the auxiliary basis set def2/J.^100^ The diamagnetic substitution method (DSM)^71,101^ was used to “switch off” the certain paramagnetic Cu^II^ centers, which were replaced with Zn^II^. The X-ray atom coordinates of **2** were used without geometry optimization, unless stated otherwise. In the respective cases, geometry optimization was performed using the BP86 functional with the TZVP basis set for the metal and SVP for all other atoms. The dummy H atoms (used for the generation of structure fragments) were generated by using the Avogadro 1.2.0 program.^102^ The energies of the high-spin and broken-symmetry states were used to extract the *J* value in copper dimers, according to the formalism *J*_AB_ = −2(*E*_HS_ – *E*_BS_)/(*S*_A_ + *S*_B_)^2^.^103−106^ The MAGPACK program was used to calculate the magnetic susceptibilities and energy states.^107^ The isosurfaces of spin densities were drawn using the VESTA 3.5.2 program.^108^

## Supporting Information Available

Additional experimental, synthetic, and spectroscopic data, details of the crystal structure studies, additional figures and tables as described in the text (PDF)

## Accession Codes

CCDC 2064900–2064901 contain the supplementary crystallographic data for this paper. These data can be obtained free of charge via www.ccdc.cam.ac.uk/data_request/cif, or by emailing data_request@ccdc.cam.ac.uk, or by contacting The Cambridge Crystallographic Data Centre, 12 Union Road, Cambridge CB2 1EZ, UK; fax: +44 1223 336033.
