# Supplementary material for: A 3D MOF based on Adamantoid Tetracopper(II) and Aminophosphine Oxide Cages: Structural Features and Magnetic and Catalytic Properties
Source: Inorg Chem. 2021 Jun 13;60(13):9631–44. doi: 10.1021/acs.inorgchem.1c00868 (PMC8277165; doi:10.1021/acs.inorgchem.1c00868)
Supplement: Supplementary file 1 — ic1c00868_si_001.pdf [file ic1c00868_si_001.pdf]

Supporting Information

A 3D MOF based on Adamantoid Tetracopper(II) and Aminophosphine Oxide Cages: Structural Features and Magnetic and Catalytic Properties

Ewelina I. Śliwa,[†] Dmytro S. Nesterov,[‡] Marina V. Kirillova,[‡] Julia Kłak,[†]

Alexander M. Kirillov[*,‡,⊥] and Piotr Smoleński[*,†]

[†]Faculty of Chemistry, University of Wrocław, F. Joliot-Curie 14, 50-383, Wrocław, Poland

[‡]Centro de Química Estrutural and Departamento de Engenharia Química, Instituto Superior Técnico, Universidade de Lisboa, Av. Rovisco Pais, 1049–001 Lisbon, Portugal

[⊥]Research Institute of Chemistry, Peoples' Friendship University of Russia (RUDN University), 6 Miklukho-Maklaya st., Moscow, 117198, Russian Federation

Table of contents

Further spectroscopic characterization of the products 3

Additional crystallographic data 3

Experimental Part (Catalytic Studies)........ 4

Figure S1. Solvent accessible voids available in the framework of the structure **2**........ 6

Figure S2. Fragment of the structure of **2** showing the difference electron density map around the O2 and O4 atoms. 6

Figure S3. EPR (X-band) spectra of powdered sample **2** at 293 and 77 K........ 7

Figure S4. FT–IR spectra for **1**........ 7

Figure S5. FT–IR spectra for **2** (route 1). 8

Figure S6. FT–IR spectra for **2’** (route 2). 8

Figure S7. TGA-DTA data for **2** (route 1). 9

Figure S8. TGA-DTA data for **2’** (route 2). 10

Figure S9. Oxidation of cyclohexane (total yield *vs* time) catalysed by **2** in the presence of H_2O_2 11

Figure S10. Oxidation of cyclohexane (yield *vs* time) catalysed by **2** in the presence of H_2O_2........ 11

Figure S11. Effect of the catalyst **2** amount on the total yield of the products and the maximum initial reaction rate 12

Table S1. Experimental and DFT calculated exchange couplings in the selected dinuclear complexes 13

Table S2. Experimental and DFT DSM calculated exchange couplings in $\{Cu_4OCl_6X_4\}$ complexes........ 14

Table S3. Singlet-quintet Δ_{12} gaps calculated for various fragments of the structure **2** using different basis sets. 15

Table S4. Calculated Δ_{ij} gaps and exchange coupling constants J compared to experimentally determined J.[a] 16

Table S5. Comparison of catalytic activity of **2** with other catalysts 17

Supporting references........ 18

Further Spectroscopic Characterization of 2

The IR spectra of **2** (Figures S6 and S7) exhibit a set of vibrations in the 1450−550 cm^{-1} range typical for PTA=O moieties, as well as characteristic band of the P=O group at 1158 and 1156 cm^{-1}, respectively. The latter is shifted in comparison to that in uncoordinated PTA=O (1164 cm^{-1}).[17-27] Other characteristic vibrations concern the weak ν(CH) bands in the 2960–2925 cm^{-1} range. The broad and intense ν(OH) bands with a maximum at 3413−3429 cm^{-1} are also observed.

Additional Crystallographic Data

In **2**, the difference map reveals numerous weak peaks around the O2 atom, which could be treated as H atoms or be an indication for a wrongly assigned atom type of O2. An attempt to treat O2 site as chlorine atom failed, showing an unacceptably large U_{eq} as compared to that for Cu2 site. Further, the theoretical powder diffractogram of this model revealed a very high intensity of the 2 2 0 reflection at 2Θ = 7.62, what contradicts to the experimental powder patterns (Figure 1). The analysis of the Cambridge Structural Database revealed that in the axially distorted fragment CuO_4Cl_2 (where *d*(Cu–O) < 2 Å and *d*(Cu–Cl) > 2.1 Å) the shortest observable Cu–Cl distance (2.69 Å; mean distance was 2.80 Å) is longer than expected Cu2–Cl distance of 2.61 Å in **2** (for the model where O2 site was treated as chlorine).

The charge balance in **2** required deprotonation of either chloroethanol (O4) or coordinated water molecules (O2). O2 atom can make four H-bonds to nearest symmetrically equivalent O4 atoms, suggesting that exact positions of O2 hydrogen atoms are hardly to determine. The difference electron density (peaks of 0.55 e Å^{-3}) nereby the O4 atom is directed toward the O2 atom (Figure S2) and could be interpreted as H atom, localized at the hydroxyl group (O4) of the chloroethanol molecule. However, the overall analysis of the electron density, as well as CSD statistics, evidences for deprotonated chloroethanol as a reliable solution. According to the CSD, the mean *d*(Cu–OH_2) distance in the axially distorted fragment $CuO_4(OH_2)_2$ is 2.47 Å. The Cu2–O2 distance in **2** (2.54 Å) falls into the range of thouse confirmed by the CSD (from 2.21 to 2.76 Å) for axially coordinated water. Only few structures containing OH group, axially coordinated to copper centre, were found in the CSD, with the mean *d*(Cu–O) distance of 2.60 Å. Also, most of the cases interpreted by CSD ConQuest as Cu–OH fragment (where O atom is set to form two bonds) are in fact Cu–ROH ones. The true cases of copper hydroxo complexes are rare.[S1]

From the X-ray analysis of **2**, the following formula can be deducted: $[Cu_4(\mu\text{-}Cl)_6(\mu_4\text{-}O)Cu(OH)_2(\mu\text{-}PTA{=}O)_4]_n \cdot 2n(Cl\text{-}EtOH) \cdot 2.5nH_2O$. This formula well agrees with the elemental analysis data (calcd.: C 22.60; H 4.47; N 11.29; MW=1488.14). For powdered sample **2’**, the formula with a slightly different

solvent composition can be deduted by elemental analysis and TG-DTA: 6n(H_2O) (calcd.: C 20.87; H 4.52; MW=1381.16). Loss of solvent and/or adsorption of air moisture upon grinding of crystals is a common effect.[S2] The homogeneity of the bulk sample **2** was confirmed by the powder X-ray technique which reveals a characteristic pattern structure at high 2*Θ* angles (Figure S3). Comparison of the theoretical powder X-ray patterns generated for complete structure of **2** and the structure with eliminated uncoordinated solvent molecules shows only little difference between them (Figure S3). The powder X-ray diffractogram of **2’** (route 2) fits with those calculated for **2**, thus suggesting that **2’** obtained by route 2 has the same $[Cu_4(\mu\text{-}Cl)_6(\mu_4\text{-}O)Cu(OH)_2(\mu\text{-}PTA{=}O)_4]_n\cdot$ framework as **2**.

Experimental Part (Catalytic Studies)

Catalytic Oxidation of Cyclohexane. Catalytic tests were performed in glass reactors equipped with a condenser under aerobic conditions and vigorous stirring at 50 °C, using acetonitrile as a solvent (up to 2.5 mL total reaction volume). Total volume refers to all reagents and solvent (H_2O_2, alkane, gas chromatography standard, H_2O, and CH_3CN). The typical procedure was as follows: catalyst **2** (2.5 µmol, used as synthesized) was introduced into a CH_3CN solution, followed by the addition of CF_3COOH promoter (TFA, optional, 0.025 mmol) and GC internal standard (CH_3NO_2, 250 µL). Then, cyclohexane (1 mmol) and hydrogen peroxide (5 mmol, 50% in H_2O) were added. The progress of the oxidation was monitored by withdrawing small aliquots of the reaction mixture at different time periods. Prior to GC (gas chromatography) analysis, the aliquots were treated with solid PPh_3 for reducing cyclohexyl hydroperoxide (primary oxidation product) and remaining H_2O_2. Generation of cyclohexyl hydroperoxide was confirmed by performing some GC analyses before and after the treatment with solid PPh_3 (Shul’pin’s method).[S3] GC peaks were attributed by comparing the chromatograms with commercially available samples of products.

Catalytic Oxidation of Propane. In a typical procedure, catalyst **2** (2.5 µmol, used as synthesized) and acetonitrile solvent (up to 2.5 mL) were introduced into a stainless-steel autoclave (20.0 mL total volume). Then, CH_3NO_2 (GC internal standard, 250 µL) and hydrogen peroxide (5 mmol 50% in H_2O, 5 mmol) were added and the autoclave was closed and pressurized with propane (1-8 atm). It was kept under stirring at 50 °C for 3-24 h (oil bath and magnetic stirrer). The autoclave was then cooled down and degassed. Samples of the reaction mixture were treated with PPh_3 and analyzed by GC for quantification of products (internal standard method).

Catalytic Carboxylation of Alkanes. In a typical procedure, catalyst **2** (1.25–2.5 µmol, used as synthesized), H_2O (2.0 mL), CH_3CN (4.0 mL), cyclohexane (1.0 mmol), and $K_2S_2O_8$ (1.50 mmol) were introduced into a stainless-steel autoclave (20.0 mL total volume). Then, the autoclave was closed and flushed three times with CO for air removal and pressurized with carbon monoxide (20 atm). In the

case of propane carboxylation, the reactor was first flushed and pressurized with propane (1 atm) followed by the addition of CO. The reaction mixture was stirred at 60 °C for 4 h (oil bath and magnetic stirrer). After this period, the autoclave was cooled in an ice bath, degassed, and opened. The reaction mixture was transferred into a glass flask. Et_2O (9.0 mL) and GC internal standard (cycloheptanone, 45 µL) were introduced. After stirring the obtained mixture for 10 min, the aliquot was taken from an organic layer and subjected to GC analysis for the quantification (internal standard method) of carboxylic acids as principal products (ketones and alcohols were also formed as by-products in minor amounts). Peaks were attributed by comparing the obtained chromatograms with those of commercially available samples of products.

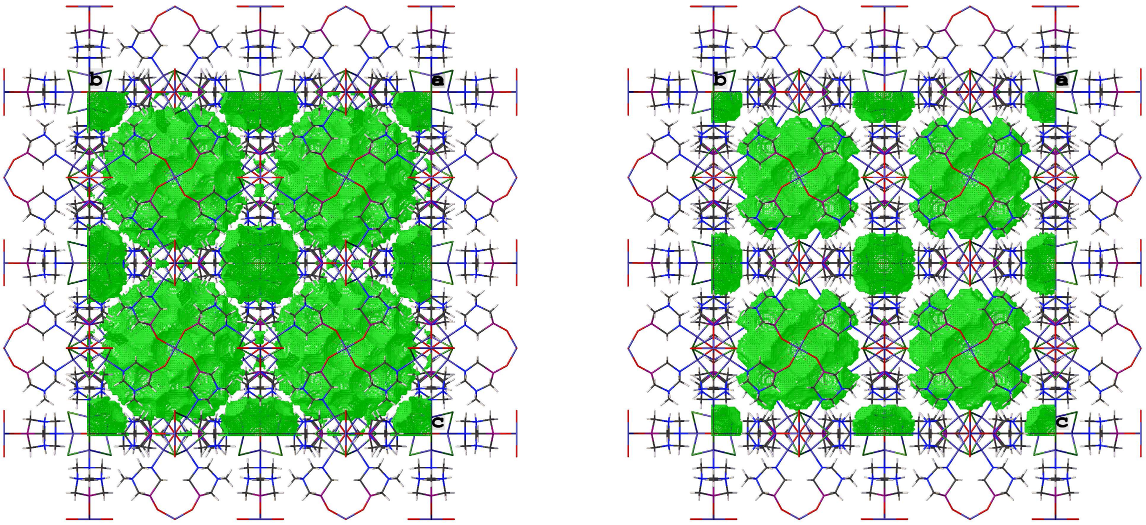

Figure S1. Solvent accessible voids available in the framework of **2** (uncoordinated chloroethanol and water molecules were eliminated) at 0.2 Å resolution, shown for 0 and 0.5 Å distance from the voids surface.

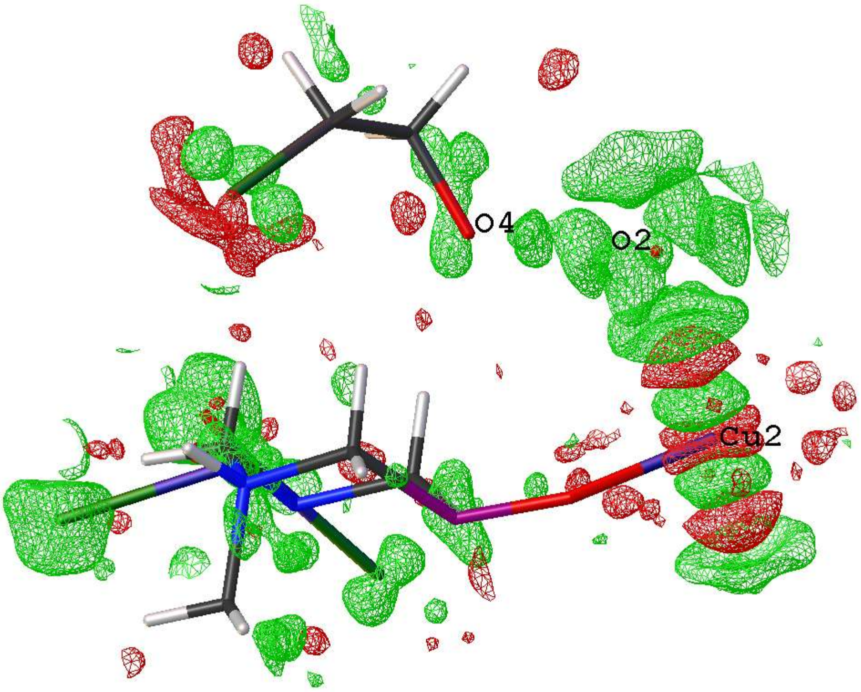

Figure S2. Fragment of the structure of **2** showing the difference electron density map at 0.5 e Å^{-3} level around the O2 and O4 atoms.

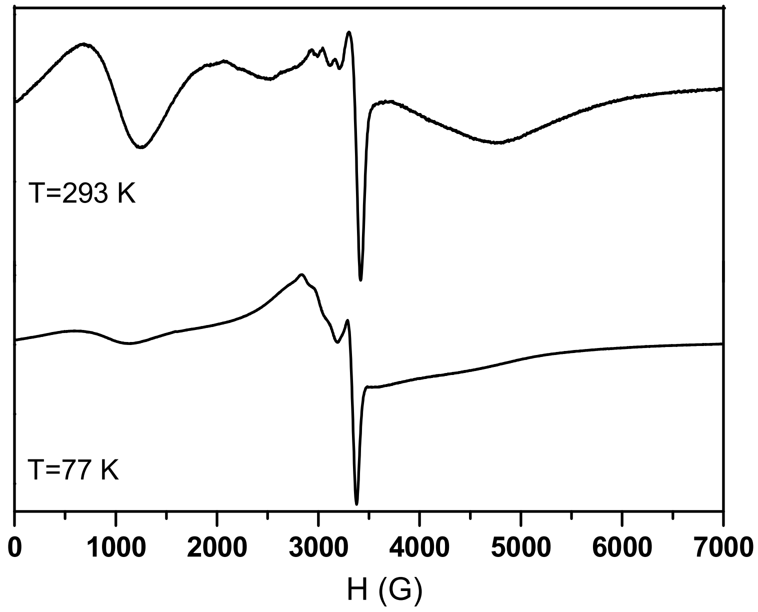

Figure S3. EPR (X-band) spectra of powdered sample **2** at 293 and 77 K.

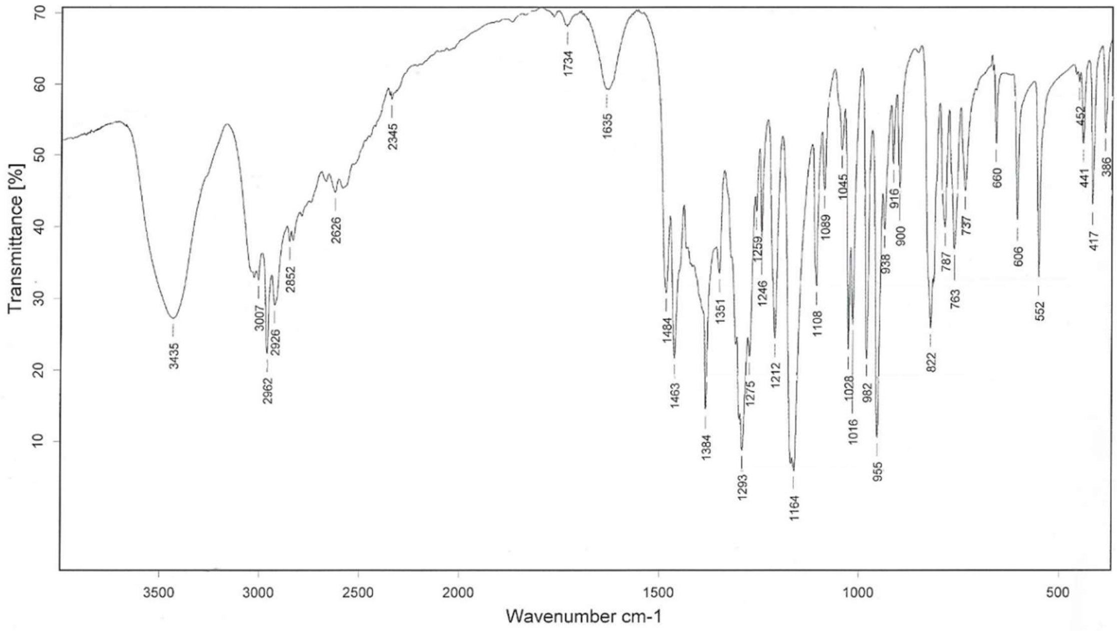

Figure S4. FT–IR spectrum of **1**.

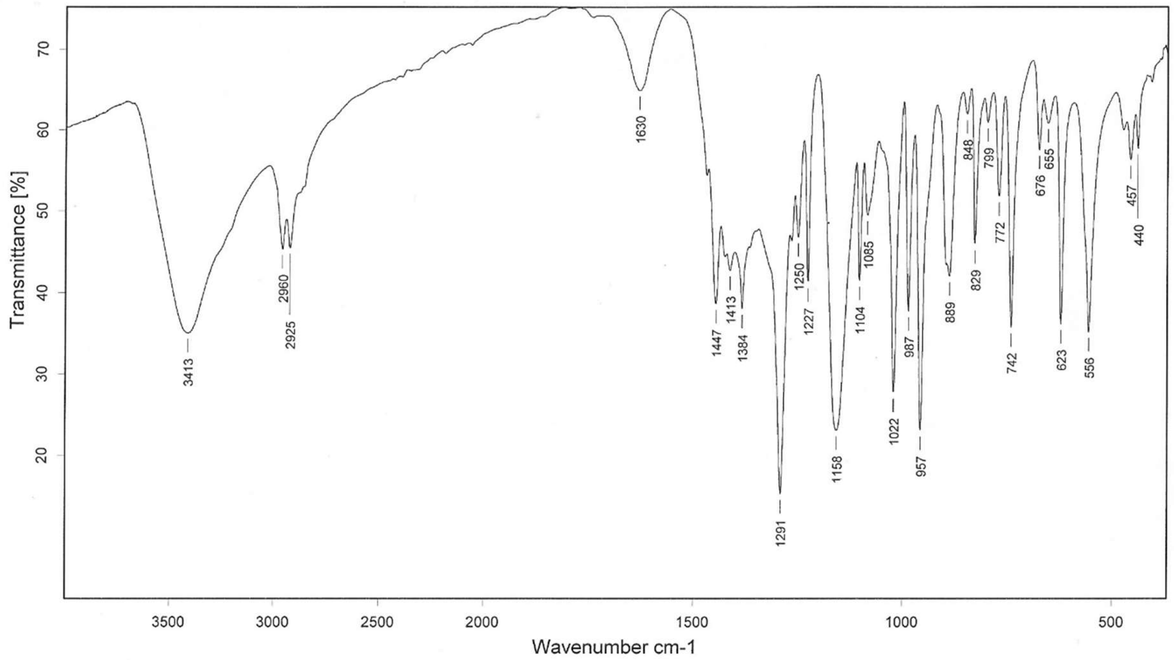

Figure S5. FT–IR spectrum of **2** (route 1).

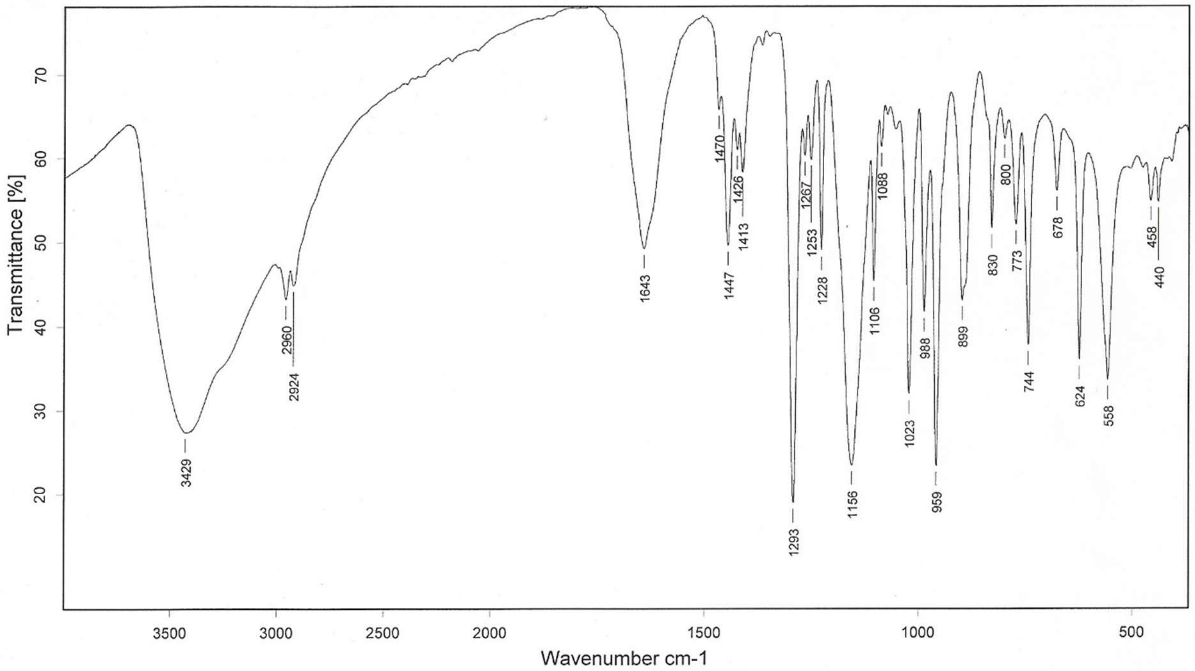

Figure S6. FT–IR spectrum of **2’** (route 2).

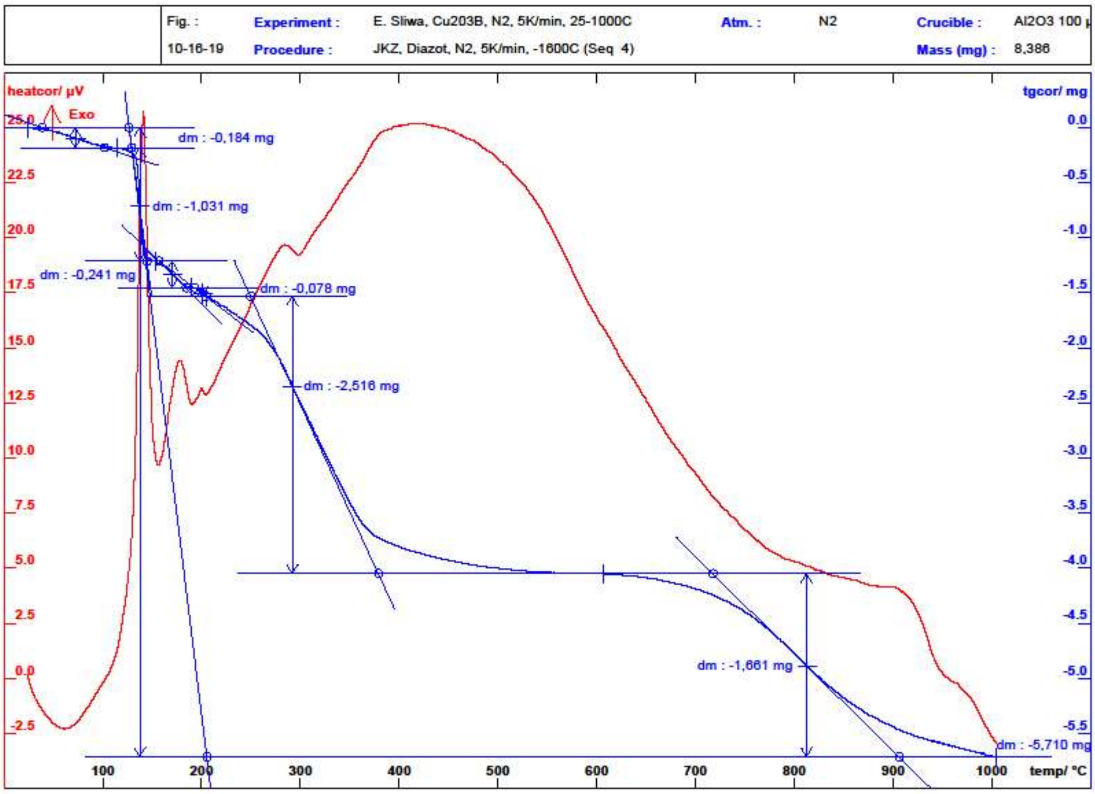

Figure S7. TGA-DTA data for **2** (route 1).

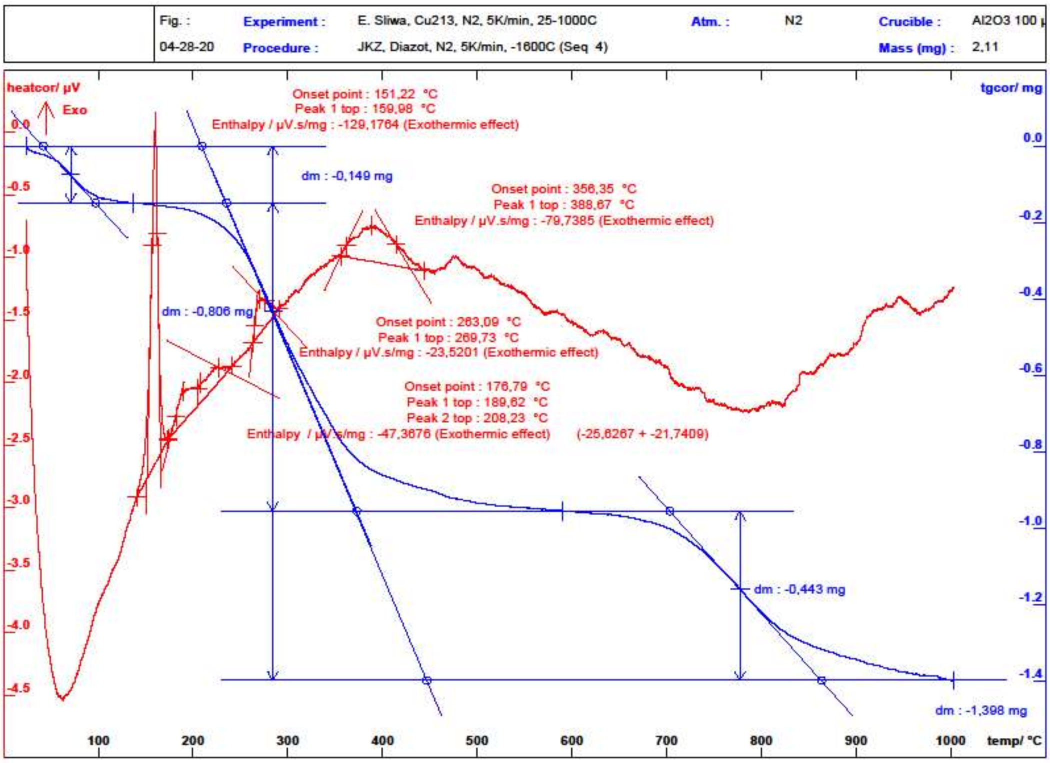

Figure S8. TGA-DTA data for **2’** (route 2).

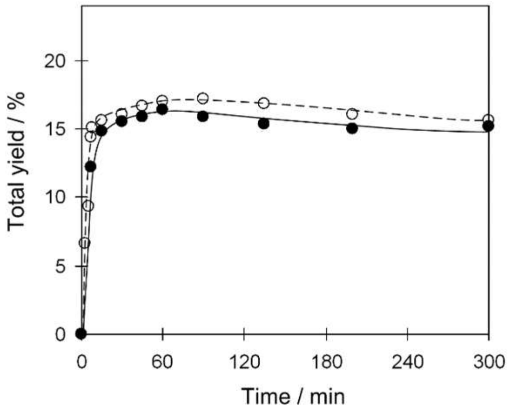

Figure S9. Oxidation of cyclohexane with H_2O_2 to a mixture of cyclohexanol and cyclohexanone (total yield *vs.* time) catalysed by **2** in the absence (dotted curve) and presence (solid curve) of the acid promoter (TFA, CF_3COOH). Reaction conditions: C_6H_{12} (1.0 mmol), H_2O_2 (50% aqueous, 5.0 mmol), **2** (2.5 µmol), TFA (optional, 0.025 mmol), CH_3CN (up to 2.5 mL total reaction volume), 50 °C.

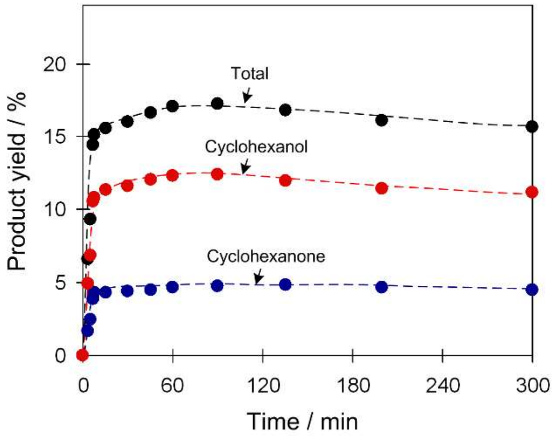

Figure S10. Oxidation of cyclohexane with H_2O_2 to a mixture of cyclohexanol and cyclohexanone (yield *vs* time) catalysed by **2.** Reaction conditions: C_6H_{12} (1.0 mmol), H_2O_2 (50% aqueous, 5.0 mmol), **2** (2.5 µmol), CH_3CN (up to 2.5 mL total reaction volume), 50 °C.

a) b)

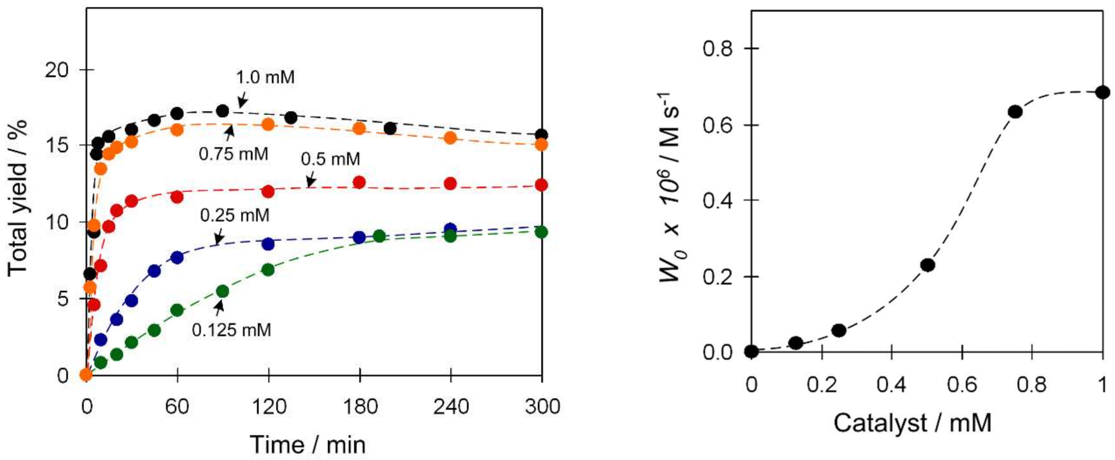

Figure S11. Effect of the catalyst **2** amount on (a) the total yield of the products (cyclohexanol and cyclohexanone, %) and (b) the maximum initial reaction rate (*W_0*) in the C_6H_{12} oxidation with of H_2O_2. Reaction conditions: C_6H_{12} (1.0 mmol), H_2O_2 (50% aqueous, 5.0 mmol), **2** (1.25-10 µmol), CH_3CN (up to 2.5 mL total reaction volume), 50 ^oC.

Table S1. Experimental and DFT calculated exchange couplings in the selected dinuclear complexes.[a]

CSD refcode	Complex	Experimental J / cm^{-1}	Calculated J / cm^{-1}	Ref.
CITLUN	$[Cu_2(OAc)(OH)Cl(bpy)_2]Cl\cdot 8.5H_2O$	145.30	162.04	S4
FESJIW	$[Cu_2(L^1)Cl](BPh_4)\cdot ac$	–670.00	–610.46	S5
FODHOV	$[Cu_2(L^2)(OH)Cl_3]\cdot DMF$	–520.00	–271.70	S6
JEDFUU	$[Cu_2(L^3)Cl_3(HOCH_3)]$	–348.00	–629.62	S7
EBEFIB	$[Cu_2(L^4)_2(O_2CH)(OH)(OCH_3)](ClO_4)$	62.50	–1.08	S8
EBEFIB_NH$_3$[b]	$[Cu_2(L^4)_2(O_2CH)(OH)(OCH_3)](ClO_4)\cdot(NH_3)_2$	62.50	–20.60	S8
RUXDIX01	$[Cu_2(L^4)_2(O_2CH)(OH)Cl](ClO_4)\cdot 0.5H_2O$	79.10	215.72	S8
YAHYUC	$[Cu_2(L^4)_2(O_2CH)(OH)Cl](PF_6)$	79.70	182.20	S9

[a] The J values were adjusted to $H = -JS_1S_2$ formalism; [b] the strong intermolecular O···H–N hydrogen bonds (d(O···N) = 2.89 Å) between the O atoms of the formate ligand and a neighbouring amino-group were modelled by adding two dummy H atoms to the H–N atoms; OAc = acetate; bpy = 2,2′-bipyridine; HL^1 = polypyridyl ligand, containing a phenolic group; ac = acetone; L^2 = ethyl substituted 3,6-bis(2-benzimidazolylthio)pyridazine; HL^3 = *S*-1,2-bis(benzimidazol-2-yl)-1-hydroxyethane; L^4 = di-2-pyridylamine.

Table S2. Experimental and DFT DSM calculated exchange couplings in $\{Cu_4OCl_6X_4\}$ complexes.[a]

CSD refcode	Complex[b]	*d*(Cu···Cu) / Å	∠(Cu–Cl–Cu) / °	∠(Cu–O–Cu) / °	Calcd. *J* / cm^{-1}	Exp. *J* / cm^{-1}	Ref.
–	**2**: $\{Cu_4OCl_6(PTAO)_4\}$	3.138	82.03	109.87	135.94	20.1	t.w.
		3.126	81.64	109.27	165.64	20.1	
–	**2**: $\{Zn_3CuOCl_6(NMe_3)_3(PTAO)$-$Cu(Me_3PO)_3(H_2O)_2\}$[c]	–	–	–	–5.30	0.04[d]	t.w.
JIWKAB	$\{Cu_4OCl_6(L^5)_4\}$[e]					$J_a = J_{12} = J_{34}$<br>$J_b = J_{13} = J_{23} = J_{24} = J_{14}$<br><br>$J_a = –35.4$<br>$J_b = –22.9$	64
		3.097	81.11	108.45	212.42		
		3.097	81.11	108.45	205.86		
		3.079	81.45	107.84	228.10		
		3.173	78.81	112.41	–95.36		
		3.173	78.84	112.41	–95.40		
		3.083	81.90	107.36	212.88		
		3.097	81.11	108.45	205.86[f]		
CUQFID	$\{Cu_4OCl_6(L^6)_4\}$					$J = J_{12} = J_{13} = J_{23} = J_{34}$<br>$J_S = J_{24}$; $J_L = J_{14}$<br><br>$J = –7.65$<br>$J_S = 83.3$; $J_L = –58.8$	63
		3.114	80.94	108.66	150.04		
		3.127	78.54	109.47	112.46		
		3.167	80.08	111.63	54.68		
		3.125	78.78	110.05	122.42		
		3.059	79.79	106.77	178.24		
		3.124	80.20	110.20	150.04		
WEXYON	$\{Cu_4OCl_6(L^7)_4\}$					$J_a = J_{12} = J_{34}$<br>$J_b = J_{13} = J_{23} = J_{24} = J_{14}$<br><br>$J_a = –1.16$<br>$J_b = –0.86$	65,66
		3.107	80.54	109.67	167.24		
		3.091	80.10	108.44	124.92		
		3.149	80.39	112.29	106.86		
		3.132	80.82	109.83	148.78		
		3.093	79.03	108.57	149.70		
		3.090	80.21	108.01	207.86		

[a] The *J* constants were calculated by the Diamagnetic Substitution Method (DSM). All *J* values were adjusted to $H = –JS_1S_2$ formalism; [b] the zinc atoms used for DSM are not stated, except of the calculation of the long-range exchange; [c] the long-range exchange coupling. PTAO ligands, except that serving as a bridge, were truncated to NMe_3 or Me_3PO ones, depending on the coordination mode; [d] *zJ* molecular field correction term; [e] phenyl groups of the ligands L[1] were substituted with methyl groups; [f] full structure of the L[5] ligand; L[5] = 3(5),4-dimethyl-5(3)-phenylpyrazole; L[6] = imidazole; L[7] = morpholine.

Table S3. Singlet-quintet Δ_{12} gaps calculated for various fragments of the structure **2** using different basis sets. The configuration used for further studies is highlighted.

Fragment	Basis sets (def2-X)		
	For metal atoms and first coordination sphere	For all other atoms	Δ_{12} / cm^{-1}
{$Cu_4OCl_6(PTA{=}O)_4$}	TZVPP	SVP	–142.650
{$Cu_4OCl_6(PTA{=}O)_4$}[a]	TZVPP	SVP	–297.489
{$Cu_4OCl_6(NMe_3)_4$}[b]	TZVPP	TZVPP	–140.868
**{$Cu_4OCl_6(NMe_3)_4$}**[b]	**TZVP**	**TZVP**	**–136.589**
{$Cu_4OCl_6(NMe_3)_4$}[b]	TZV	TZV	–63.378
{$Cu_4OCl_6(NMe_3)_4$}[b]	SVP	SVP	–198.536
{$Cu_4OCl_6(NH_3)_4$}	TZVPP	TZVPP	56.306
{$Cu_4OCl_6(NH_3)_4$}[a]	TZVPP	TZVPP	–143.709
{$Cu_4OCl_6(NH_3)_4$}	SVP	SVP	–24.752
{$Cu_4OCl_6(NH_3)_4$}	SVP	SVP	–214.549

[a] after geometry optimization; [b] the chain-of-spheres approximation (RIJCOSX) used.

Table S4. Calculated Δ_{ij} gaps and exchange coupling constants J compared to experimentally determined J.[a]

Complex or CSD Refcode	Spin configuration	Δ_{ij} / cm^{-1}	J / cm^{-1} Direct[b]	L.s. fit[c]	Constrained l.s. fit[d]	Experimentally determined	Ref.
**2:** $\{Cu_4OCl_6(NMe_3)_4\}$	βααα	90.17	23.44	32.07	32.27	$J = J_{12} = J_{13} = J_{23} = J_{34} = J_{24} = J_{14}$	t.w.
	αβαα	93.28	23.94	32.67	31.87		
	ααβα	91.72	23.85	32.50	32.27		
	αααβ	92.54	22.79	31.41	31.87	J = 20.1	
	ββαα	136.57	22.01	30.64	31.87		
	βαβα	137.88	23.97	32.78	31.87		
	αββα	137.13					
FEVYAH	βααα	100.60	36.11	35.95	54.25	$J_a = J_{12} = J_{23} = J_{34} = J_{14}$	76
	αβαα	117.02	72.83	72.67	54.25	$J_b = J_{13} = J_{24}$	
	ααβα	132.28	50.93	50.74	54.25		
	αααβ	114.98	55.96	55.84	54.25	J_a = 65	
	ββαα	145.40	8.99	8.73	4.94	J_b = –1	
	βαβα	214.89	8.55	8.31	4.94		
	αββα	103.65					
CUQFID	βααα	–42.32	–1.29	14.26	–	$J = J_{12} = J_{13} = J_{23} = J_{34}$	63
	αβαα	33.61	–14.09	6.78	–	$J_S = J_{24}$; $J_L = J_{14}$	
	ααβα	–8.98	–4.04	–3.28	–		
	αααβ	–5.24	–50.18	–26.01	–	J = –7.65	
	ββαα	–6.14	–21.83	–11.64	–	J_S = 83.3; J_L = –58.8	
	βαβα	–7.64	18.00	–24.34	–		
	αββα	52.80					

[a] B3LYP/def2-TZVP for all atoms, accompanied with RIJCOSX approximation. The literature J values were adjusted to $H = -J_1J_2$ formalism used herein; [b] Direct determination of J values from the combination of Δ_{ij} gaps, according to Ruiz approach (7); [c] J values obtained as a result of the least-square fit of the J_{ij} set to the Δ_{ij} one using the relations (6), performed by minimizing the R-factor, where $R = \sum_n \frac{(\Delta_{n,calcd} - \Delta_{n,exp})^2}{\Delta_{n,exp}^2}$; [d] J values obtained through the procedure described in [c], additionally using the symmetry constrains: $J_{12} = J_{34}$ and $J_{13} = J_{23} = J_{24} = J_{14}$.

Table S5. Comparison of catalytic activity of **2** with other catalysts in mild oxidation and carboxylation of alkanes.

Catalyst[b]	Oxidation	Carboxylation	Reference
	Substrate (total product yield)	Substrate (total product yield, %)	
$[Cu_4(\mu\text{-}Cl)_6(\mu_4\text{-}O)Cu(OH)_2(\mu\text{-}PTA{=}O)_4]_n$ (**2**)	Propane (14%, TON 280)	Propane (28%)	This work
$[Cu_4(\mu\text{-}Cl)_6(\mu_4\text{-}O)Cu(OH)_2(\mu\text{-}PTA{=}O)_4]_n$ (**2**)	Cyclohexane (17%)	Cyclohexane (32%)	This work
$[Cu_4(\mu\text{-}Hbes)_3(\mu\text{-}H_2bes)(\mu\text{-}L)]$[c]	Propane (7-9%, TON 60-77)[a]	Propane (28-40%)	S10
$[Cu_4(\mu\text{-}Hbes)_3(\mu\text{-}H_2bes)(\mu\text{-}L)]$[c]	Cyclohexane (8-13%)[a]	Cyclohexane (40-43%)	S10
$[Fe(\mu_3\text{-}Hcpna)_2]_n$[d]	Propane (7%)[a]	Propane (17%)	S11
$[Fe(\mu_3\text{-}Hcpna)_2]_n$[d]	Cyclohexane (15%)[a]	Cyclohexane (21%)	S11
$[Cu_2(\mu\text{-}Hbdea)_2(\mu\text{-}bdca)]_n$[e]	Cyclohexane (18%)[a]	Cyclohexane (43%)	S12

[a]Only active in the presence of acid promoter. [b]Simplified formulae without solvent of crystallization are given. [c]HL = benzoic, 4-hydroxybenzoic, or 3-hydroxybenzoic acid. [d]H_2cpna = 5-(4'-carboxyphenoxy)nicotinic acid. [e]H_2bdea = N-butyldiethanolamine, H_2bdca = 4,4'-biphenyldicarboxylic acid.

Supporting References

[S1] Farrokhpour, H.; Hadadzadeh, H.; Darabi, F.; Abyar, F.; Rudbari, H. A.; Ahmadi-Bagheri, T. A Rare Dihydroxo Copper(II) Complex with Ciprofloxacin; a Combined Experimental and ONIOM Computational Study of the Interaction of the Complex with DNA and BSA. *RSC Adv*. **2014**, *4*, 35390-35404.

[S2] Nesterov, D. S.; Kokozay, V. N.; Dyakonenko, V. V.; Shishkin, O. V.; Jezierska, J.; Ozarowski, A.; Kirillov, A. M.; Kopylovich, M. N.; Pombeiro, A. J. L. An Unprecedented Heterotrimetallic Fe/Cu/Co Core for Mild and Highly Efficient Catalytic Oxidation of Cycloalkanes by Hydrogen Peroxide. *Chem. Commun*., **2006**, 4605–4607.

[S3] Shul'pin, G. B. New Trends in Oxidative Functionalization of Carbon–Hydrogen Bonds: A Review. *Catalysts* **2016**, *6*, 50; (b) Shul'pin, G. B. Metal-Catalyzed Hydrocarbon Oxygenations in Solutions: Thedramatic Role of Additives: a Review. *J. Mol. Catal. A: Chem.* **2002**, *189*, 39−66.

[S4] Youngme, S.; Phatchimkun, J.; Wannarit, N.; Chaichit, N.; Meejoo, S.; van Albada, G. A.; Reedijk, J. New Ferromagnetic Dinuclear Triply-Bridged Copper(II) Compounds Containing Carboxylato Bridges: Synthesis, X-ray Structure and Magnetic Properties. *Polyhedron* **2008**, *27*, 304-318.

[S5] Karlin, K. D.; Farooq, A.; Hayes, J. C.; Cohen, B. I.; Rowe, T. M.; Sinn, E.; Zubieta, J. Models for Met-Hemocyanin Derivatives: Structural and Spectroscopic Comparisons of Analogous Phenolate and X (X = OH^-, OMe^-, N_3^-, Cl^-, OAc^-, OBz^-) Doubly Bridged Dinuclear Copper(II) Complexes. *Inorg. Chem.* **1987**, *26*, 1271-1280.

[S6] Thompson, L. K.; Mandal, S. K.; Rosenberg, L.; Lee, F. L.; Gabe, E. J. Binuclear Copper(II) Complexes of Some Tetradentate (N_4) Diazine Ligands with Benzimidazole Donor Groups. Crystal Structure of [μ-3,6-Bis(N-Ethyl-2-Benzimidazolylthio)Pyridazine-N, μ-N^5, μ-N^6, N]-(μ-Hydroxo)(μ-Chloro)Dichlorodicopper(II) DMF. *Inorg. Chim. Acta* **1987**, *133*, 81-91.

[S7] van Albada, G. A.; Mutikainen, P.; Turpeinen, U.; Reedijk, J. Crystal Structure, Magnetism and Spectroscopy of a Dinuclear Cu(II) Compound with a Chiral Ligand; $Cu_2(SL)(\mu\text{-}Cl)(Cl)_2(CH_3OH)$ (HSL = S-1,2-Bis(Benzimidazol-2-yl)-1-Hydroxyethane). *Polyhedron* **2006**, *25*, 81-86.

[S8] Youngme, S.; Chailuecha, C.; Van Albada, G. A.; Pakawatchai, C.; Chaichit, N.; Reedijk, J. Synthesis, Crystal Structure, Spectroscopic and Magnetic Properties of Doubly and Triply Bridged Dinuclear Copper(II) Compounds Containing Di-2-Pyridylamine as a Ligand. *Inorg. Chim. Acta* **2004**, *357*, 2532-2542.

[S9] Youngme, S.; Chailuecha, C.; Van Albada, G. A.; Pakawatchai, C.; Chaichit, N.; Reedijk, J. Dinuclear Triply-Bridged Copper(II) Compounds Containing Carboxylato Bridges and Di-2-Pyridylamine as a Ligand: Synthesis, Crystal Structure, Spectroscopic and Magnetic Properties. *Inorg. Chim. Acta* **2005**, *358*, 1068-1078.

[S10] Costa, I. F. M.; Kirillova, M. V.; André, V.; Fernandes, T. A.; Kirillov, A. M. Tetracopper(II) Cores Driven by an Unexplored Trifunctional Aminoalcohol Sulfonic Acid for Mild Catalytic C–H Functionalization of Alkanes. *Catalysts*, **2019**, *9*, 321.

[S11] Zhao, N.; Li, Y.; Gu, J.; Kirillova, M.V.; Kirillov, A.M. Synthesis, Structural Features, and Catalytic Activity of an Iron(II) 3D Coordination Polymer Driven by an Ether-Bridged Pyridine-Dicarboxylate. *Crystals* **2019**, *9*, 369.

[S12] Kirillova, M. V.; Fernandes, T. A.; André, V.; Kirillov, A. M. Mild C−H Functionalization of Alkanes Catalyzed by Bioinspired Copper(II) Cores. *Org. Biomol. Chem.*, **2019**, *17*, 7706-7714.
